# Structural Design and Immunogenicity of a Novel Self‐Adjuvanting Mucosal Vaccine Candidate for SARS‐CoV‐2 Expressed in Plants

**DOI:** 10.1111/pbi.70278

**Published:** 2025-07-21

**Authors:** Mi‐Young Kim, Andy Cano Tran, Ju Kim, Humblenoble Stembridge Ayuk, Adam Sparrow, Lorenzo Bossi, Megan Brown, Emil Joseph Vergara, Kathrin Göritzer, Elisabetta Groppelli, Tae‐Ho Kwon, Julian K. C. Ma, Yong‐Suk Jang, Rajko Reljic

**Affiliations:** ^1^ Department of Molecular Biology and the Institute for Molecular Biology and Genetics Jeonbuk National University Jeonju Republic of Korea; ^2^ Institute for Infection and Immunity City St George's University of London London UK; ^3^ Université Claude Bernard Lyon 1 Villeurbanne France; ^4^ ImmunXperts SA A Q^2^ Solutions Company Charleroi Belgium; ^5^ Gencellbiotech Inc. Wanju Republic of Korea

**Keywords:** APCs targeting, cholera toxin B subunit (CT‐B), immunoglobulin Fc, macro molecular adjuvant, mucosal vaccine platform, SARS‐CoV‐2

## Abstract

Mucosal vaccination for COVID‐19 to boost preexisting though insufficient systemic and local/mucosal immunity remains an attractive prospect but there are currently no licensed mucosal vaccines against this infection. Here, using a plant expression system, we developed a novel mucosal vaccine platform for respiratory viruses and demonstrated its application in the context of SARS‐CoV‐2 infection. In addition to the antigen itself, the PCF (Platform CTB‐Fc) vaccine candidate incorporates two molecular adjuvants, the IgG‐Fc antibody fragment and the nontoxic cholera toxin B subunit (CTB), with the first targeting the vaccine to IgG receptors on antigen‐presenting cells, and the second providing local adjuvanticity by targeting cellular gangliosides in the mucosae. We demonstrated that this vaccine candidate is highly immunogenic in mice, inducing virus‐neutralising systemic and mucosal antibodies as well as tissue resident memory T cells in the lungs. We also demonstrated that SRBD‐PCF is recognised by immune cells from exposed or vaccinated individuals, and that circulating antibodies also bind to the antigen within the vaccine, forming immune complexes (IC). Finally, with a view of respiratory delivery, we demonstrated that the vaccine can be aerosolised without loss of material or biological activity, and that it is noncytotoxic and nonhaemolytic to human cells. Furthermore, we demonstrate that the plant expression system represents a suitable platform to produce these complex, multifunctional macromolecules capable of simultaneously binding to multiple targets. Our data strongly support the case for a safe, self‐adjuvanting mucosal COVID‐19 vaccine development, as means to boosting both systemic and mucosal immunity.

## 1 Introduction

Highly pathogenic beta coronaviruses of recent times evolved from the Severe Acute Respiratory Syndrome Coronavirus (SARS‐CoV) in 2002 (Volz et al. 2021) through the Middle East Respiratory Syndrome Coronavirus (MERS‐CoV) in 2012 (Al‐Osail and Al‐Wazzah 2017) and emerged as a threat to human health and public safety in late 2019 all over the world as SARS‐CoV‐2 (Zhu et al. 2020). While WHO declared the end of COVID‐19 as a health emergency in May 2023 (Cheng et al. 2023) the virus still circulates in the community, adapting less virulent forms that can survive in the human population probably for a very long time (Chen et al. 2022). This underscores the need for ongoing efforts to develop effective vaccines against SARS‐CoV‐2 and improve strategies for rapid responses to similar diseases in the future.

The spike (S) glycoprotein of SARS‐CoV‐2, which is the primary target for vaccine development, is made up of two subunits: S1, containing the receptor binding domain (RBD), and S2, which facilitates membrane fusion. This glycoprotein forms trimers on the surface of virions (Ke et al. 2020). However, variants of SARS‐CoV‐2 continue to emerge (Chen et al. 2020; Campbell et al. 2021; Liu et al. 2022; Cele et al. 2022) with many mutations occurring in the S protein, a main target of neutralising antibodies in coronavirus infections. Therefore, it is of utmost importance to emphasise the careful selection of virus antigens to avoid/reduce variants, but also to fine‐tune vaccine formulations, so that they elicit balanced Th1 and Th2 responses instead of Th17 responses, which are correlated with severe adverse effects post‐vaccination (Martonik et al. 2021). The current vaccine platforms, including live/attenuated vaccines, mRNA, viral vectors, virus‐like particles (VLPs), DNA, and protein vaccines, have successfully reduced deaths and serious illness from SARS‐CoV‐2 (Sachs et al. 2022), but the virus still remains in circulation and poses a risk for new variants causing further outbreaks. These highlight the need for improved vaccine formulations or additional vaccine strategies, especially those that could potentially not only reduce the severity of disease but also potentially prevent early viral replication and therefore reduce transmission.

The development of protein‐based vaccines, both licensed and in preclinical stages, requires external adjuvants. However, these are not easily accessible, have limited human applicability, and can raise safety concerns, contributing to vaccine scepticism or hesitancy. Despite the importance of mucosal immunity in fighting respiratory diseases, progress in developing mucosal vaccines has been slow, and most are limited to the live attenuated platform, like the Flu Mist for influenza (Carter and Curran 2011). Mucosal vaccines based on other platforms, including recombinant protein subunits, have not yet been approved. In this study, we propose using a macro‐size protein‐based vaccine platform that simultaneously targets ganglioside GM_1_ on epithelial cell surfaces and Fc‐IgG receptors on antigen‐presenting cells (APC) to combat respiratory diseases such as SARS‐CoV‐2. This technology eliminates the need for external adjuvants, which represents a significant departure from conventional protein vaccine development, and instead, the vaccine generates its own adjuvanticity by antigen fusion to the non‐toxic cholera toxin B subunit (CTB) and the Fc portion of Immunoglobulin G (platform CTB‐Fc; PCF), with both components acting as molecular adjuvants. We previously demonstrated the immunogenicity of the PCF vaccine platform in the context of dengue infection (Kim, Vergara, et al. 2024), and now for the first time, demonstrate its potential as a mucosal vaccine for a respiratory infection by incorporating SARS‐CoV‐2 Spike protein Receptor Binding Domain into PCF (SRBD‐PCF) and testing the vaccine in vitro and in vivo. Furthermore, we demonstrate the production feasibility of these self‐adjuvanting macro‐sized proteins by a cost‐effective plant expression system, achieving reasonable production yields and purity in a laboratory setting.

## 2 Material and Methods

### 2.1 Gene Construction and Prediction of Structure

To generate SRBD‐PCF of SARS‐CoV‐2 (isolated from Wuhan‐Hu‐1) corresponding to S protein amino acid sequence 321–521 (based on NCBI reference sequence: NC_045512.2 or UniProtKB—P0DTC2) fused to human/mouse PCF, a similar protocol was used as published previously (Kim, Vergara, et al. 2024). PCF contains two major components: N‐terminal CTB (UniProtKB—Q8LT24_9VIRU) and C‐terminal human IgG1‐Fc (SRBD‐hPCF) for ex vivo human studies or mouse IgG2a‐Fc (SRBD‐mPCF) for mouse experiments, with the SRBD antigen sandwiched in between. The modified human and mouse Fc sequences used in this study are available in the Supporting Information of a previously published article (https://doi.org/10.1111/pbi.12741) (Kim, Vergara, et al. 2024). SRBD‐PCF was designed to accumulate in the endoplasmic reticulum (ER) by inclusion of the N′‐terminal signal peptide (Sack et al. 2007) and C′‐terminal ER retention peptide. The genes, optimised for plant codon usage and tagged with *NcoI* restriction enzyme sites followed by 5′ UTR‐signal peptide of rice amylase 3D gene at the 5′ end, and *XbaI* restriction enzyme sites following the ER retention hexapeptide (SEKDEL) and stop codon at the 3′ end, were synthesised by Invitrogen GeneArt Gene Synthesis Services. The fragment of SRBD‐PCF digested with *NcoI‐XbaI* was inserted into the pTRAk.2 plant expression vector (Sack et al. 2007). Prediction of protein structure was generated using the ColabFold and ChimeraX package. All programs used in this study are listed in Supporting Information Method S1.

### 2.2 Expression, Purification Biophysical Characterisation of SRBD‐PCF

To express SRBD‐PCF in tobacco (*Nicotiana benthamiana*), the plasmid DNA was transformed into *Agrobacterium tumefaciens* strain GV3101 containing pMP90RK helper plasmid by electroporation. Subsequently, it was transiently expressed in plant cells using the vacuum infiltration method, as previously described (Kim, Vergara, et al. 2024). Protein extracts from the infiltrated leaves (5–6 days after) and purification were performed also as previously described (Kim, Vergara, et al. 2024), with steps including affinity chromatography using a protein A agarose affinity column (Sigma‐Aldrich), protein concentration using Amicon Ultra Centrifugal Filter (100 kDa MWCO, Millipore) and buffer exchange by dialysis in PBS (pH 7.4). To increase protein stability during aerosolisation, 0.05% polysorbitol‐80 (Tween‐80) was added to the final preparation. For size fractionation, SRBD‐PCF protein was subjected to size‐exclusion chromatography (SEC) on a HiLoad 16/600 Superdex 200 pg column (GE Healthcare, USA) equilibrated with PBS pH 7.4 using an ÄKTA pure (GE Healthcare, USA) FPLC system. Various analytical assays were then performed to characterise the purified vaccine construct. Biophysical assessment (Coomassie) staining and Western blotting procedures are described in Method S2. Molecular size measurement of SRBD‐PCF scaffold by dynamic light scattering (DLS) and SEC analyses is described in Method S3. Functional in vitro assessment of SRBD‐hPCF, including assays for complement C1q, GM_1_ ganglioside, and ACE2 binding by ELISA, is described in Method S4, while the binding to APC by flow cytometry and internalisation assays by confocal microscopy are described in Method S5. Binding of SRBD‐PCF to human tonsillar mononuclear cells (TMC) is described in Method S6. The feasibility of aerosolisation and mucosal stability of SRBD‐PCF protocols is described in Method S7, while the assay for potential cytotoxicity to human cells is described in Method S8.

### 2.3 Human PBMC/DC Coculture and T Cell Proliferation

For peripheral blood mononuclear cell (PBMC) assays, human blood from volunteers who were SARS‐CoV‐2 infected, uninfected, or vaccinated with or without prior infection was used to test if SRBD could induce their proliferation in vitro. Uninfected‐unvaccinated donor served as a baseline control. For this purpose, dendritic cells (DC) were first generated from PBMC for co‐culturing with autologous T cells from the donors (Method S9). Total CD4+ T cells were isolated from PBMCs of the same donors with magnetic separation (EasySep Human CD4+ T Cell Enrichment Kit, StemCell). T cells were then co‐cultured with DC that had been stimulated with SRBD‐hPCF and their proliferation assessed after 5 days by measuring 5‐Ethynyl‐2′‐deoxyuridine (EdU) incorporation. EdU is a thymidine analogue which is incorporated into the DNA of dividing cells during the S‐phase. Cell cultures were pulsed with 1 μM EdU for approximately 16 h. The next day, cells were fluorescently stained for viability and T cell surface markers (CD3‐BV421, Biolegend and CD4‐FITC, Miltenyi), fixed, permeabilised, and the incorporated EdU was stained with a fluorescent azide (Click‐iT EdU Flow Cytometry Assay Kit, Invitrogen, Thermo fisher Scientific). Cells were then acquired on a BD Fortessa X‐20.

### 2.4 Detection of SRBD‐hPCF by Human Sera of COVID‐19 Vaccines

To test if SRBD antigen within polymeric SRBD‐hPCF construct is accessible to circulating antibodies (and therefore B cells), donor sera from COVID‐19 vaccinated people were used in indirect ELISA, Western blot and C1q binding ELISA. For indirect ELISA, 10 μg/mL SRBD‐hPCF was coated into 96‐well plates, and human serum samples (1/50) were 3‐fold serially diluted followed by anti‐human kappa IgG‐HRP (1/2500) as the secondary antibody for detection of human immunoglobulins. For Western blot analyses, 1 μg of antigens including plant‐derived SRBD‐hPCF, SRBD‐hFc, and mammalian‐derived RBD, purified from Expi293F cells, using constructs as described in (Goritzer et al. 2024) were separated on the SDS‐PAGE gel, transferred to nitrocellulose (Western) and incubated with sera (1/400), followed by detection with anti‐human kappa IgG‐HRP (1/2500). For C1q binding ELISA, 1 μg/mL of SRBD‐hPCF was pre‐incubated with 10 μg/mL purified human IgG from seropositive donors (by Protein G affinity chromatography) at 37°C and the assay developed as described above. Human naïve serum from Sigma (H4522) was used as the negative control.

### 2.5 Immunisation of Mice and Sample Collection

Six–Eight‐week‐old female BALB/c mice purchased from Charles River were maintained under specific pathogen‐free conditions. 10 μg of SRBD and the antigen‐equivalent of the mouse version of SRBD‐mPCF (25 μg) were used per dose. A total of nine mice were allocated per group, with five mice from each group tested for initial antibody responses before further immunisations. All groups received three doses of the relevant vaccine candidate at 2‐weekly intervals. Groups were as follows: PBS (G1); SRBD‐mPCF without adjuvant (only nasal immunisations for G2 and subcutaneous priming (G3) followed by subcutaneous or nasal boosting for G3‐1 and G3‐2, respectively); SRBD‐mPCF with Quil‐A adjuvant subcutaneous priming followed by nasal boosting (G4); and SRBD with Quil‐A adjuvant by the same regimen as for G4 (G5). Quil‐A is a saponin‐based compound, extracted from the bark of the *Quillaja saponaria* tree and commonly used as a commercially available adjuvant to stimulate the immune system. At the end of the immunisation regimen, mice were anaesthetised under isoflurane and cardiac puncture was performed to collect blood. Bronchoalveolar lavage fluid (BALF) was collected from the lungs of culled mice by injecting 1 mL of sterile PBS into the lungs via an incision in the trachea followed by three rounds of flushing. The washes were then centrifuged at 500 rcf and the supernatant was collected and stored at −20°C until further use, while the cellular fraction was separated for analysis of tissue‐resident T cells (TRM). For the measurement of antigen‐specific IgA and IgG, BALF was concentrated 10‐fold using Amicon 50 kDa centrifugal concentrator tubes (Millipore). Antibody responses in sera and BALF were measured by ELISA as described in Methods S10. Spleens were collected, disintegrated into culture medium and used in cytokine secretion assays as described previously (Kim, Vergara, et al. 2024), and in Method S11.

### 2.6 Analyses for TRM From Lungs and BALF by Flow Cytometry

Single cell suspensions of 4 million cells per sample for lung homogenates and the entire cellular fraction of BALF were transferred to 96‐well U‐bottom plates and centrifuged at 500 rcf for 5 min. Cells were washed with sterile PBS three times, then stained using a cocktail of antibodies (1:200) including mouse anti‐CD3 (APC), CD4 (PerCP/Cy5.5), CD8 (BV510), CD44 (FITC), CD62L (PE), CD69 (PE/Cy7), CD103 (BV421) (Biolegend, USA), fixable viability marker (1:500) (Invitrogen), and mouse Fc block (TruStain fcXTM anti‐mouse CD16/32) (1:250) (Biolegend) for 30 min at 4°C. Stained cells were then washed twice and resuspended in sterile 1× PBS prior to FACS (Fluorescence‐Activated Cell Sorting) acquisition on the CytoFlex (Beckman Coulter) and analysis using FlowJo V10 software.

### 2.7 Generation of SARS‐CoV‐2 Pseudovirus and Neutralisation Assay

To generate SARS‐CoV‐2 pseudovirus, HEK293T cells were grown to 60%–80% confluency in Dulbecco's Modified Eagle Medium (DMEM; Sigma‐Aldrich) supplemented with 10% foetal bovine serum (FBS), penicillin (100 U/mL), and streptomycin (100 μg/mL). Cells were then transfected with a mixture of plasmids p8.91 (encoding gag, pol, and rev), pCSFLW (encoding luciferase), and pCAGGS‐SARS‐CoV‐2 Spike using the X‐tremeGENE 360 system according to the manufacturer's instructions. Supernatants containing pseudovirus were harvested, filtered through a 0.45 μm filter, and stored at −80°C until use.

Serum samples from experimental animals were heat‐inactivated at 56°C for 30 min prior to use in the assay. BALF samples were concentrated tenfold using a 3 kDa cut‐off centrifugal concentrator column (Merck Millipore) and used without heat inactivation and further dilutions. Serum samples were then 2‐fold serially diluted (1:40 to 1:5120) in OptiMEM (Thermo Fisher Scientific) and added to a 96‐well tissue culture plate. Pseudovirus was added to each well at a final concentration of 2 × 10^7^ RLU/mL. The serum‐pseudovirus mixture was then incubated for 1 h at 37°C, 5% CO_2_.

HEK293T cells expressing ACE2 and TMPRSS2 (Genecopeia) were grown in DMEM and prepared by trypsin dissociation, washing with PBS, and resuspending in fresh DMEM media. A total of 200 000 cells were added to each well, and the plates were incubated for 48 h at 37°C, 5% CO_2_, prior to the addition of the pseudo virus for 1 h. Luciferase activity was then measured using Bright‐Glo reagent (Promega) according to the manufacturer's instructions. The relative light units (RLU) were read on a plate reader (BMG Omega). Results were normalised to the pseudovirus‐only control and expressed as a percentage of inhibition, calculated as 1—(normalised value).

### 2.8 Immunisation and SARS‐CoV‐2 Virus Challenge in hACE2 KI Mice

The viral challenge study was performed at the animal facility at the Jeonbuk National University in Korea, under the local ethical approval and licence. 8–14‐week‐old hACE2‐All CDS B6J knock‐in (hACE2 KI) mice (Cyagen) as a challenge model were immunised subcutaneously with 50 μg of RBD (mixed with 15 μg of Quil‐A) as prime (to mimic exposed or vaccinated individuals). Four weeks after the first immunisation, mice were boosted intranasally twice at 2‐week intervals with 6 μg of RBD or 15 μg of SRBD‐mPCF (molar equivalent), and PBS was used as a negative control. To assess antigen‐specific antibody responses, serum was collected 3 days after the last boost. Additionally, to evaluate SARS‐CoV‐2 viral load, immunised mice were inoculated via the nasal route with 2 × 10^5^ focus‐forming units (FFUs) of SARS‐CoV‐2 (Wuhan strain, S clade, and NCCP 43326) 2 weeks after the last boost. On Day 8, specimens of lung tissue were collected from infected mice. The gene expression of the N protein of SARS‐CoV‐2 in lung tissue was measured by quantitative real‐time reverse transcription‐polymerase chain reaction (qRT‐PCR, Method S12).

### 2.9 Ethics Statement

Detection of antigen with COVID‐19 vaccinated blood, T cell stimulation with SRBD‐PCF pulsed DCs using COVID‐19 infected/vaccinated blood from donors was performed under the ethical protocol/amendment IXP‐001_V3 (Belgium; Reg. Nr. B6702014215858), protocol IXP‐003_V1 (Belgium; Reg. Nr. B707201627607). Assays with TMC were performed under ethical approval from St George's Hospital (ethical approval REC Ref No 18/SC/0203). Mouse studies of the immunogenicity of SRBD‐PCF were performed at the biological research facility at London School of Hygiene & Tropical Medicine under Home Office animal project licence 70/7490, while the SARS‐CoV‐2 pathogenic challenge study was performed at Jeonbuk National University (JBNU) in accordance with the Korean Animal Protection and Welfare Division Regulations under the Ministry of Agriculture, Food and Rural Affairs.

### 2.10 Statistics

Statistical analyses were performed using GraphPad Prism 7. One‐way ANOVA multi‐comparison test followed by Sidak's or Tukey's post hoc correction test was performed to determine statistically significant differences between various conditions, as defined by *p* ≤ 0.05. Confidence levels are indicated by single or multiple asterisks, as indicated in figures. Bars represent means of biological or technical repeats and error bars represent standard error or deviation of the mean, as indicated. Specific circumstances are described in detail in Sections 3 and 4.

## 3 Results

### 3.1 Gene Construct and Prediction of Structure of SRBD‐PCF

**FIGURE 1:**
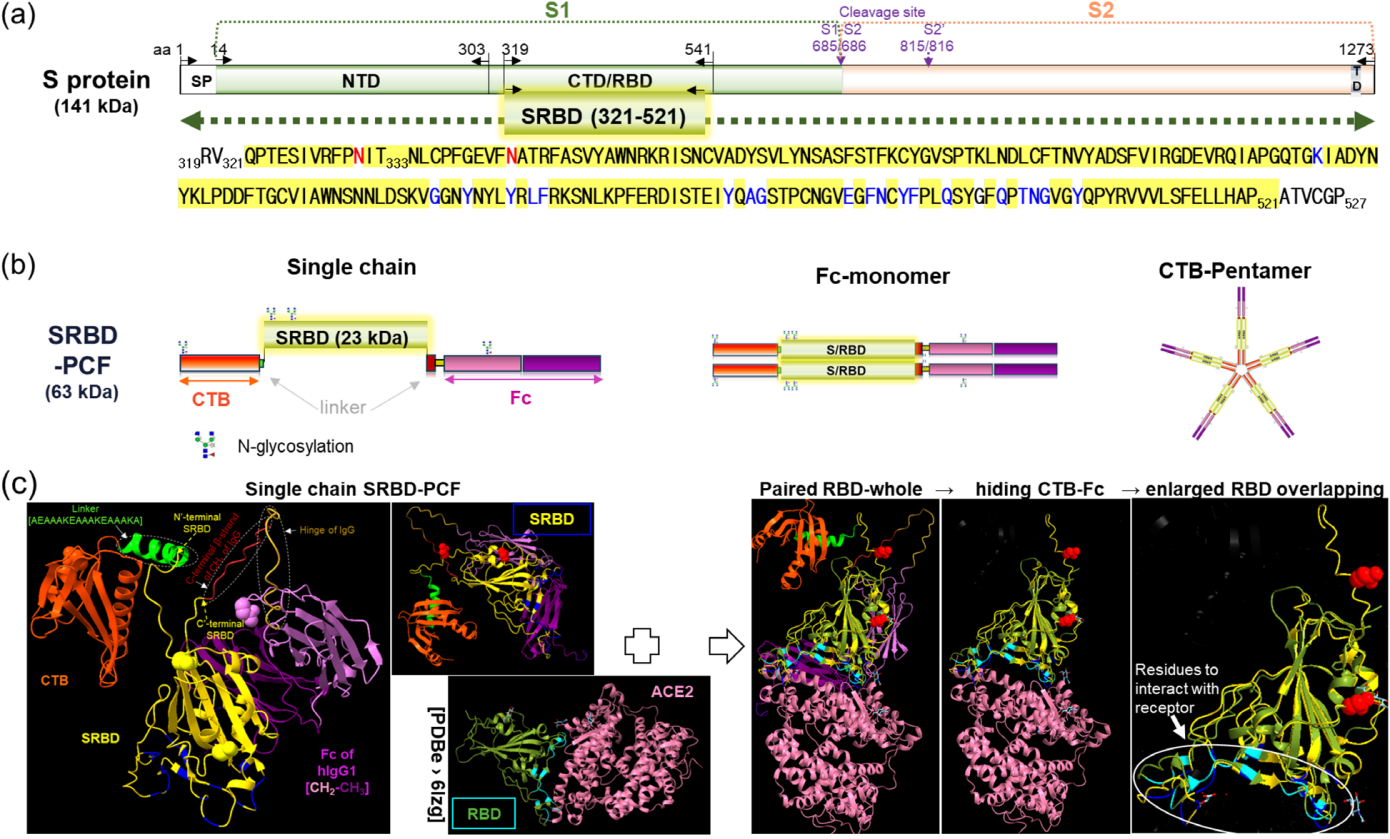
Spike protein and SRBD fused to PCF vaccine platform (SRBD‐PCF). (a) Schematic illustration of the complete spike protein sequence of SARS‐CoV‐2 (Wuhan strain) comprising S1‐S2. SP: Signal peptide; NTD (aa 14–303): N‐terminal domain; CTD/SRBD (aa 319–541): C‐terminal domain or receptor binding domain of spike protein; TD (aa 1214–1234): Transmembrane domain. The SRBD321‐521aa sequence highlighted in yellow box was incorporated into PCF. The red residues of SRBD represent two N‐glycosylation sites and blue residues that interact with hACE2, respectively. (b) Diagram of SRBD‐hPCF showing single chain, Fc‐monomer and CTB‐pentamer. Positions of the four functional N‐glycosylation sites within the single chain of PCF are indicated. (c) Prediction of SRBD‐hPCF structure (human version only) using AlphaFold 2 via UCSF ChimeraX. The red, yellow and pink/blue spheres represent functional N‐glycosylation sites in each domain and the blue stubs in the SRBD (yellow) represent the residues for interaction with hACE2 receptor as shown in panel (a). The SRBD in single chain PCF paired with RBD of the coronavirus spike receptor‐binding domain complexed with its receptor ACE2 (X‐ray diffraction: PDBe > 6lzg DOI: https://doi.org/10.2210/pdb6lzg/pdb) and zoomed‐in version (far‐right panel) of the interaction, indicating overlap between predicted and experimental models of the interaction. Linker sequences [AEAAAKEAAAKEAAAKA] are depicted in green; arrows for N′‐terminal SRBD and C′‐terminal SRBD in yellow; C‐terminal β‐strand of CH1 of IgG in brown; Hinge of IgG in gold colour presented in panel c.

To evaluate the potential of the PCF mucosal vaccine platform, we have undertaken a proof‐of‐concept study by incorporating into it the RBD of the ancestral Wuhan strain of SARS‐CoV‐2. Two hundred and one amino acids (aa) length of RBD from the S protein of SARS‐CoV‐2, termed SRBD_(321–521)_ (Figure 1a, yellow highlighted sequence). SRBD contains two functional N‐linked glycosylation sites (N331 and N343), which are important for the correct folding of the protein and for antibody recognition (Walls et al. 2020). To enhance antigen uptake by APC, SRBD was fused to the PCF vaccine backbone, generating SRBD‐PCF, and incorporating two potent molecular adjuvants CTB and IgG‐Fc at the N' and C'‐terminus, respectively. Then, SRBD‐PCF can be assembled into a polymeric form first through Fc‐monomer formation through a disulfide bond within the hinge region of the IgG heavy chain and then CTB‐pentamerisation through noncovalent bonds (Merritt et al. 1994) (Figure 1b). As the linker between CTB and the antigen, a long rigid helical structure [AEAAAKEAAAKEAAAKA] or short flexible [GPGPGS] sequence was employed for human PCF (hPCF) or mouse PCF (mPCF), respectively. To maintain the quaternary structure of IgG‐Fc, which is crucial for interactions with FcγRs, we extended it with the C‐terminal β‐strand of CH1 and the hinge region.

**FIGURE 2:**
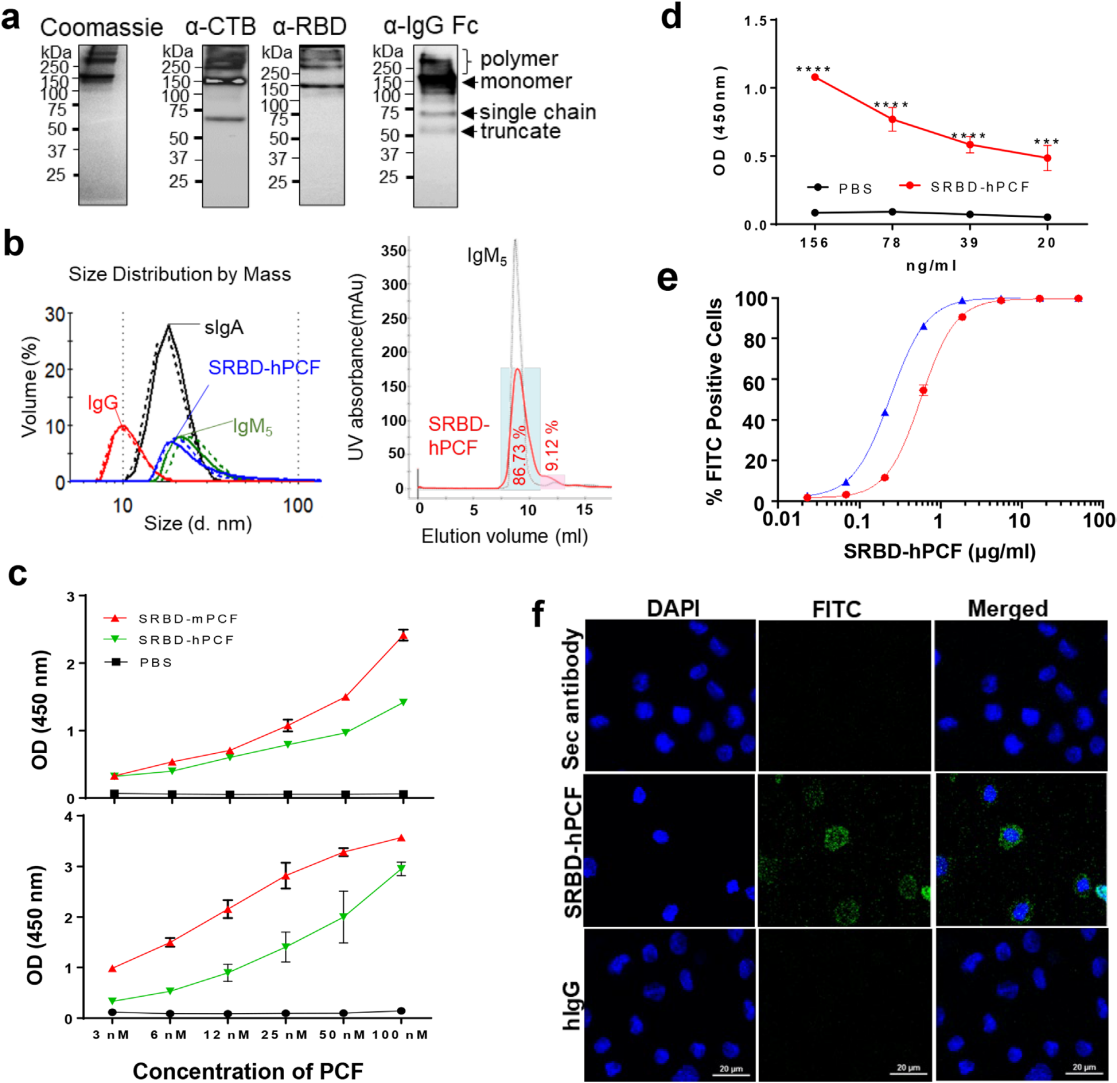
Expression and in vitro functional characterisation of plant‐derived SRBD‐hPCF. (a) Analysis of heat‐treated human version of SRBD‐PCF by SDS–PAGE under non‐reducing conditions: Coomassie staining (5 μg) and immuno‐detection (Western blotting, 0.5 μg) with anti‐CTB, anti‐RBD and anti‐hIgG Fc specific antibodies. (b) Molecular size measurement of SRBD‐hPCF by dynamic light scattering (left), and SEC analysis (right) in comparison with human IgG, sIgA and pentameric IgM. The dashed and solid lines in each sample represent two technical repeats. SEC analysis was performed in comparison with pentameric IgM, with blue box indicating 86.7% of the loaded protein content. The approximate size of SRBD‐hPCF was calculated from standard proteins curve. (c) Mouse and human SRBD‐PCF interaction with GM1 ganglioside via CTB (top panel) and C1q via Fc (bottom panel). (d) SRBD‐hPCF binding to ACE2 by ELISA; (e) concentration‐dependent SRBD‐hPCF binding to surface of macrophage/monocyte THP‐1 and U937 cells by flow cytometry. (f) Internalisation of SRBD‐hPCF by THP‐1 cells by confocal microscopy. Shown are representative samples for internalisation of SRBD‐hPCF, human IgG (as internal control) and secondary antibody alone (as negative control). ***p ≤ 0.001, ****p ≤ 0.0001.

The structure of single chain and monomeric SRBD‐PCF protein predicted by computational modelling for human SRBD‐hPCF using AlphaFold 2 via UCSF ChimeraX as previously described (Mirdita et al. 2022) is shown in Figure 1c and further detail for both human and mouse versions is given in Figure S1a,b. The single chains of SRBD‐hPCF consist of three components represented in different colours (CTB, orange, IgG‐Fc, purple; SRBD, yellow) and include linkers, N‐glycosylation sites (sphere shape) and ACE2 receptor binding residues (Wang et al. 2020) (blue stubs in yellow‐highlighted domain in Figure 1a). To evaluate the confidence level of prediction, predicted aligned errors (PAE) and predicted Local Distance Difference Test (pLDDT) were presented in the structure of the single chain and Fc‐monomer (Figure S1a,b), indicating a high level of confidence. Using the tool for matchmaker in ChimeraX software, the computational model of SRBD‐hPCF was compared to the experimental X‐ray diffraction model of RBD_319‐527aa_ sequence docking with ACE2, which previously provided the structural basis of the virus‐receptor interactions (Wang et al. 2020). The results indicated a high level of similarity (sequence alignment score = 985.2 for human PCF (Figure 1c)). For the 119 selected atom pairs (those that are likely conserved or structurally important regions) within human PCF, the Root Mean Square Deviation (RMSD) value was 0.916 Å, indicating that these parts of the two protein structures were very similar. RMSD is the most commonly used quantitative measure of the similarity between two superimposed atomic coordinates (Kufareva and Abagyan 2012), and typically, RMSD below 1 Å signifies high structural similarity. This is best illustrated in the far‐right panel in Figure 1c, which shows significant overlap between model‐generated SRBD within the fusion protein and the experimental model of RBD in complex with ACE2, which was subsequently demonstrated experimentally in Figure 2d by showing that SRBD‐hPCF binds to ACE2. In summary, SRBD in the computational model of SRBD‐hPCF and RBD in the experimental model were highly similar in structurally critical regions, indicating that the antigen is likely correctly structured within the vaccine construct and therefore available to immune cells in a biologically relevant and accessible form.

### 3.2 Expression and Functional Characterisation of SRBD‐PCF In Vitro

The molecular weight (MW) of a single chain of SRBD‐PCF is predicted at 63 kDa, not accounting for the four N‐glycosylation sites (one in CTB and Fc each, and two in SRBD, respectively). It has been reported previously that the plant‐derived RBD‐Fc carrying the ER retention peptide mainly displayed high mannose type glycan structures (Srisangsung et al. 2024), so the single chain SRBD‐PCF is therefore expected to be 70 kDa, the monomer 140 kDa and the pentamer 700 kDa, with intermediate forms (dimer to pentamer) ranging in between. The purified assembled monomeric and polymeric structures determined by denaturing SDS‐PAGE under nonreducing conditions and subsequent Coomassie staining and Western blotting by probing with domain‐specific antibodies are shown in Figure 2a. Coomassie staining revealed a prominent protein band corresponding to the monomer (by antibody Fc arrangement analogy) and several polymeric forms that were beyond the range of the largest protein standard (> 250 kDa). Western blotting also indicated the presence of polymeric forms (> 250 kDa) and the monomer (140 kDa), as well as some single chains detected with anti‐CTB and anti‐IgG but not anti‐RBD antibodies. To further characterise the molecular size but under nondenaturing conditions, the size distribution was measured by DSL and SEC analyses, in comparison with size‐known reference proteins such as human IgG (150 kDa), sIgA (320 kDa), and pentameric IgM (900 kDa). Size distribution by mass, as measured by Zetasizer, indicated that SRBD‐hPCF was bigger than human sIgA or monomeric IgG but smaller than pentameric IgM (Figure 2b, left panel). These measurements corroborated those seen by SEC analysis, with SRBD‐hPCF appearing slightly smaller than pentameric IgM and indicating an approximate Mw of 770 kDa when calculated from the standard protein curve (Figure 2b, right panel). The highlighted blue area of fractionated SRBD‐hPCF in Figure 2b contained 86.73% of the total protein, indicating that under nondenaturing conditions, the majority of the protein was assembled in pentameric form.

### 3.3 C1q, Ganglioside GM_1_ , ACE2 and APC Binding Assays

To confirm CTB activity and evaluate immune‐complex properties of SRBD‐PCF polymers, GM_1_ ganglioside, ACE2, and C1q complement component binding analyses were performed by ELISA. The assembled polymers bound to GM_1_ (Figure 2c upper panel) ganglioside and C1q complement component (Figure 2c bottom panel) in a dose‐dependent manner, while commercial IgG or secondary detection antibody could not bind. However, the efficiency of GM_1_ and C1q binding of human PCF that incorporates a rigid linker was somewhat lower in both assays than that of the mouse counterpart, which incorporates a flexible linker (Figure S2) (Kim, Vergara, et al. 2024). SRBD‐hPCF also bound to ACE2 in a concentration‐dependent manner (Figure 2d), validating our computational predictions. We then tested the capacity of SRBD‐hPCF to bind to the surface of APC using human monocyte/macrophage cell lines THP‐1 and U937. These cells were tested beforehand for expression of FcγRs and ACE2, and we found that both cell lines express CD64 (FcγR1) and CD32 (FcγRII), as well as ACE2, but not CD16 (FcγRIII), with levels of expression for all receptors higher in THP‐1 than U937 cells (Figure S3). Consequently, we observed a somewhat higher level of binding of SRBD‐hPCF to THP‐1 cells compared to U937 (Figure 2e). Internalisation of SRBD‐PCF by THP‐1 cells was demonstrated by confocal microscopy (Figure 2f), whereas monomeric human IgG could not be internalised. Altogether, these assays demonstrated that SRBD‐hPCF was assembled correctly and retained the binding capacity for GM_1_ and APC receptors, subsequently enabling antigen uptake by APCs.

### 3.4 Ex Vivo Human Studies

**FIGURE 3:**
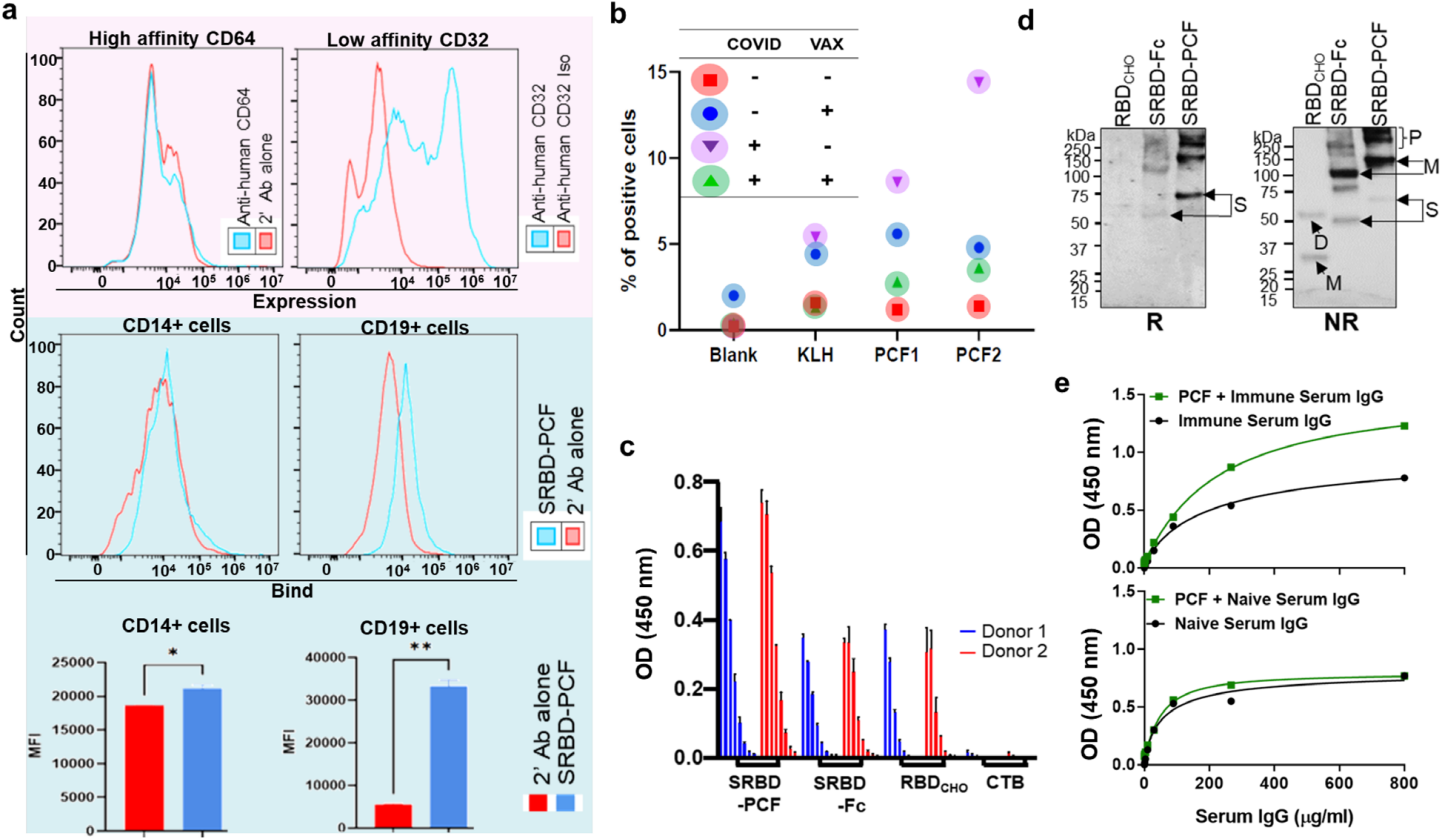
Ex vivo human studies. (a) Receptor expression and SRBD‐hPCF binding to naive tonsillar mononuclear cells (TMCs). The expression of FcγRs (CD64 and CD32) in TMCs (pink box) and SRBD‐hPCF binding to whole TMC or CD14+ and CD19+ cells (blue box) were examined; CD14+ and CD19+ were used as markers for monocytes/macrophages and B‐lymphocytes, respectively. CD14 + cells or CD19 + cells with the statistically different Mean Fluorescence Intensity (MFI) were recorded *p < 0.05, **p < 0.001. (b) Antigen recall CD4+ T cell responses by PBMC derived from COVID‐19 exposed, vaccinated (or both) or non‐exposed individuals; shown are percentages of cells proliferating in response to SRBD‐hPCF at two different concentrations (PCF1 = 5 μg/mL and PCF2 = 20 μg/mL). Each colour indicates an individual donor. ‘Blank’ is medium‐alone stimulation and KLH (Keyhole limpet haemocyanin) is positive control stimulus. (c) Detection of SRBD‐hPCF with COVID‐19 vaccinated human sera from two immunised donors. Shown are 3‐fold serial sera dilutions (starting from 1:50) binding to equivalent antigen concentrations within SRBD‐hPCF, SRBD‐hFc (no CTB) or RBD alone; CTB was also used as the negative control. (d) Immuno‐blotting SRBD‐hPCF with immune human sera from two different donors, shown for reducing or nonreducing conditions. S, single chain; M, monomer: D, dimer; P, polymer (e) C1q binding ELISA to test whether pre‐existent anti‐RBD antibodies in sera could form IC with SRBD‐hPCF and activate complement. The upper panel shows increased binding to C1q by SRBD‐hPCF and vaccinated serum IgG in a dose dependent manner, compared to vaccinated serum IgG alone. SRBD‐hPCF and naive serum IgG used as an internal control (bottom panel) show no reactivity above the baseline.

As the intended application of our vaccine platform is for mucosal delivery, and to better predict how SRBD‐hPCF may interact with primary immune cells in humans, we furthered our experiments using tonsil mononuclear cells (TMC) isolated from these secondary lymph organs before COVID‐19 onset. It was of particular interest to test for expression of IgG receptors that might be targets for SRBD‐hPCF binding. TMC showed only negligible levels of CD64 (high affinity IgG receptor) but high levels of CD32 (low affinity IgG receptor) expression (Figure 3a). In addition, there was a double peak for CD32 expression possibly indicating two distinct cell subsets bearing this receptor. When testing for binding of SRBD‐hPCF to gated CD14+ myeloid and CD19+ B cell populations by flow cytometry, we observed more efficient binding to B cells. Thus, the data showed that SRBD‐hPCF could bind to subsets of TMC, providing evidence that it could be trapped by mucosal immune cells for subsequent priming of B and T cell responses.

To test antigenicity and accessibility of SRBD displayed within the PCF construct to immune cells and antibodies, we acquired PBMC and sera from individuals who were either exposed or unexposed to SARS‐CoV‐2, with or without prior vaccination. Following stimulation with SRBD‐hPCF, we observed T cell proliferation in either exposed or vaccinated individuals, but not in unvaccinated unexposed donors (Figure 3b), indicating that the human version of the construct can be effectively taken up by endogenous APCs, processed, and presented to T cells, inducing their proliferation. We then used sera from COVID‐19 vaccinated donors to see if antibodies can bind to the SRBD moiety within PCF, as a proxy for accessibility to B cells. ELISA wells were coated with the same antigen concentration of either RBD expressed in CHO cells, RBD‐IgG‐Fc (without CTB) or SRBD‐hPCF, and probed with human sera. We detected binding for both RBD alone and RBD‐IgG‐Fc, but surprisingly, the strongest binding was for SRBD‐hPCF (Figure 3c), probably due to the higher structural arrangement of the antigen in polymeric constructs. No binding was detected when wells were coated with CTB alone, serving as a negative control.

Furthermore, when the same sera were used to test binding by Western blotting, a similar pattern was observed, with the strongest binding detected for SRBD‐hPCF and the weakest for monomeric RBD expressed in CHO cells (Figure 3d). Finally, the accessibility of the antigen to B cells and the antibodies was demonstrated in yet another assay in which we measured immune complex formation between SRBD‐hPCF and circulating anti‐SARS‐CoV‐2 antibodies by their complement C1q binding. While IgG from both naïve and COVID‐19 vaccinated individuals could bind C1q, significant additional binding was only detected when SRBD‐hPCF was combined with immune but not nonimmune IgG (Figure 3e). These experiments suggest that the antigen within SRBD‐hPCF vaccine constructs is fully accessible to human B cells and antibodies.

### 3.5 Immunogenicity of SRBD‐PCF in Mice

**FIGURE 4:**
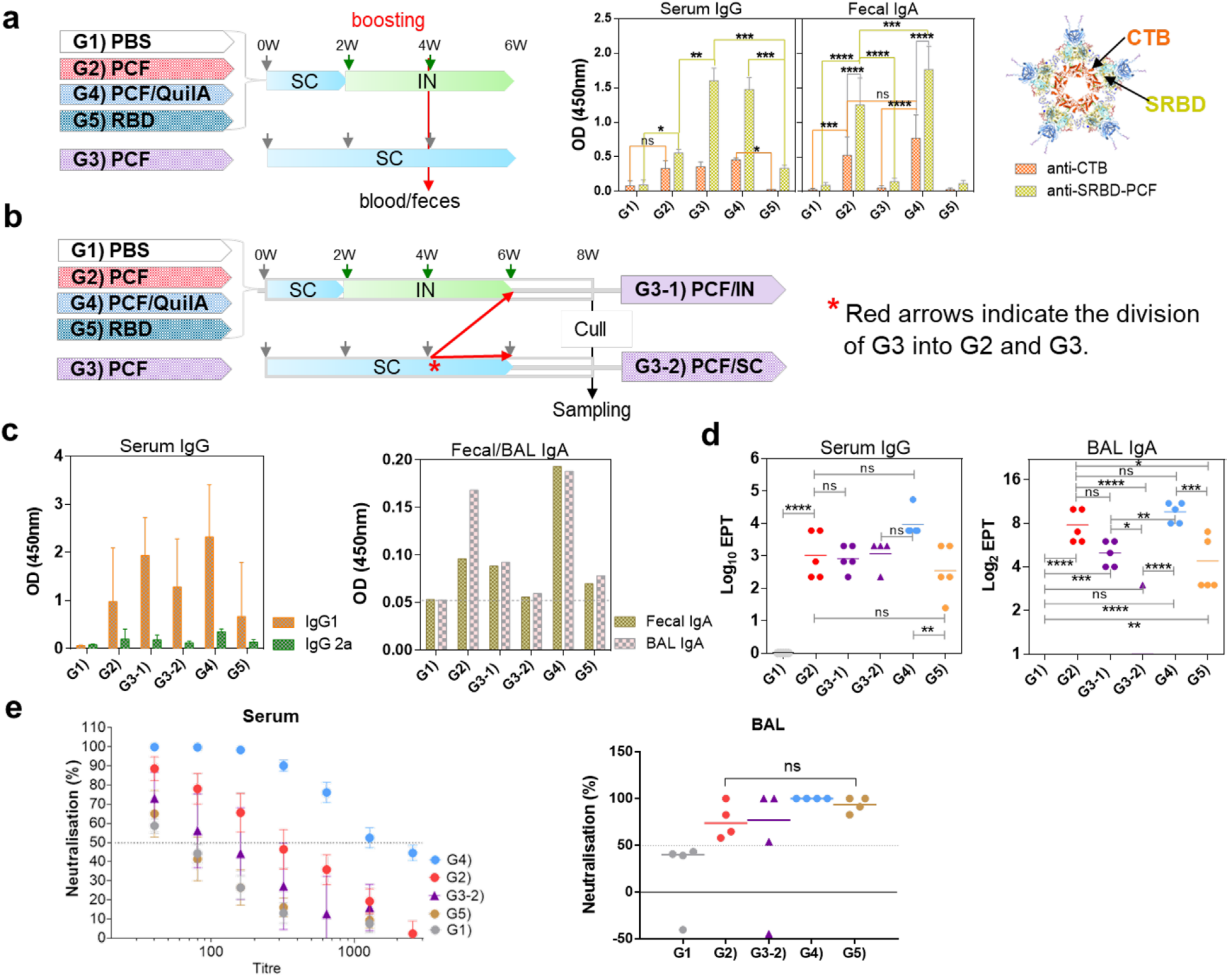
Immunogenicity of SRBD‐mPCF in mice and virus neutralisation activity. (a) Initial schedule for immunisation (*n* = 9) and antigen specific antibody responses in each group (G1–G5). Following the first boost immunisation, sera were analysed for responses against CTB and SRBD. Pooled sera IgG and faecal IgA were detected at 1:600 and 1:2 dilutions, respectively. (b) Final immunisation schedule after splitting G3 into G3‐1 (sc‐sc‐in; *n* = 5) and G3‐2 (sc‐sc‐sc; *n* = 4) (c) RBD‐specific antibody responses in sera and BALF. Detection of IgG1 and IgG2a in 1: 2000 dilutions of sera and IgA in 1:2 dilution of faeces or BALF. (d) EPT, endpoint titres for IgG in sera and IgA BALF. (e) Neutralisation ability of sera (*n* = 5) and BAL (*n* = 3 ~ 4) against pseudo CoV‐2 virus; Sera were 2‐fold serially diluted starting from 1 in 40, while BAL was tested only as single 10× concentrated sample. The dotted line indicates 50% neutralisation capacity. Statistical analysis was performed by two‐way ANOVA, followed by Sidak's multiple comparisons test. *p ≤ 0.05, **p ≤ 0.01, ***p ≤ 0.001, ****p ≤ 0.0001.

To test the immunogenicity of SRBD‐mPCF in mice, the mouse version of PCF equivalent to 10 μg of RBD was injected systemically and followed by two boosts by two different routes (nasally and subcutaneously), as indicated in Figure 4a. Antibody responses were initially measured in both blood and faeces after the first boost vaccination. As shown in Figure 4a, CTB and SRBD specific IgG and IgA antibody responses were detected in sera and faeces, respectively. Notably, the PCF groups boosted nasally without (G2) or with adjuvant (G4) induced IgA antibodies against both CTB and SRBD in faecal samples, which were not detected for mice boosted systemically (G3) or immunised with RBD alone (G5). However, nasal boosting with SRBD‐mPCF alone (G2) failed to induce a strong IgG response systemically, as seen after systemic boosting in G3 or nasal boosting but with adjuvant (G4). We hypothesised that this was due to insufficient priming of the immune response, since the mucosal route is generally thought to be more effective as a boosting strategy and may require a sufficiently primed systemic response to be fully effective. Thus, a subset of mice in G3 (G3‐1) which were twice immunised systemically were then boosted intranasally with SRBD‐mPCF, while the remainder of the mice proceeded to final systemic boosting (Figure 4b). At the end of the vaccination regimen, all mice were culled and various tissues collected for immunological analyses.

All groups of mice except those given PBS (control) induced high levels of IgG1 and low levels of IgG2a in serum (Figure 4c), indicating predominantly a Th2 type response. Highest responses in sera were observed for the adjuvanted group (G4) and the two systemic and one nasal vaccination regimen in G3‐1, while the lowest response was observed for the RBD alone group (G5). Highest faecal and BALF IgA responses were detected in the two groups that received two nasal boosts (G2, without adjuvant and G4, with adjuvant). This pattern was also reflected in end‐point titres (EPT) for both sera IgG and BALF IgA (Figure 4d). Thus, the BALF IgA EPT for individual mice in the nasally boosted G2 and G4 groups were 64 ~ 1024 and 256 ~ 2048, respectively, and higher than in any other group. Taken together, this shows that SRBD‐mPCF either alone or when combined with an adjuvant could be used to mucosally boost a pre‐existing systemic immune response and elevate antibody levels in both mucosal and systemic compartments.

Neutralisation ability of sera against pseudo CoV‐2 was found to be highest in G4, followed by G2, with serum from the RBD alone group (G5) exhibiting lowest activity and comparable to the PBS group (G1). 50% neutralising activity in sera was thus 1/1050 and 1/280 in G4 and G2, respectively, while all other groups were lower (Figure 4e). Due to the dilute nature of BALF, we only tested virus neutralising activity in concentrated samples (without further dilutions) and observed that the PBS group (G1) had no neutralising activity, and that surprisingly, all vaccine groups could neutralise similarly, including the RBD alone group (G5) (Figure 4e, right panel).

### 3.6 Cellular Responses Induced by mSRBD‐PCF

**FIGURE 5:**
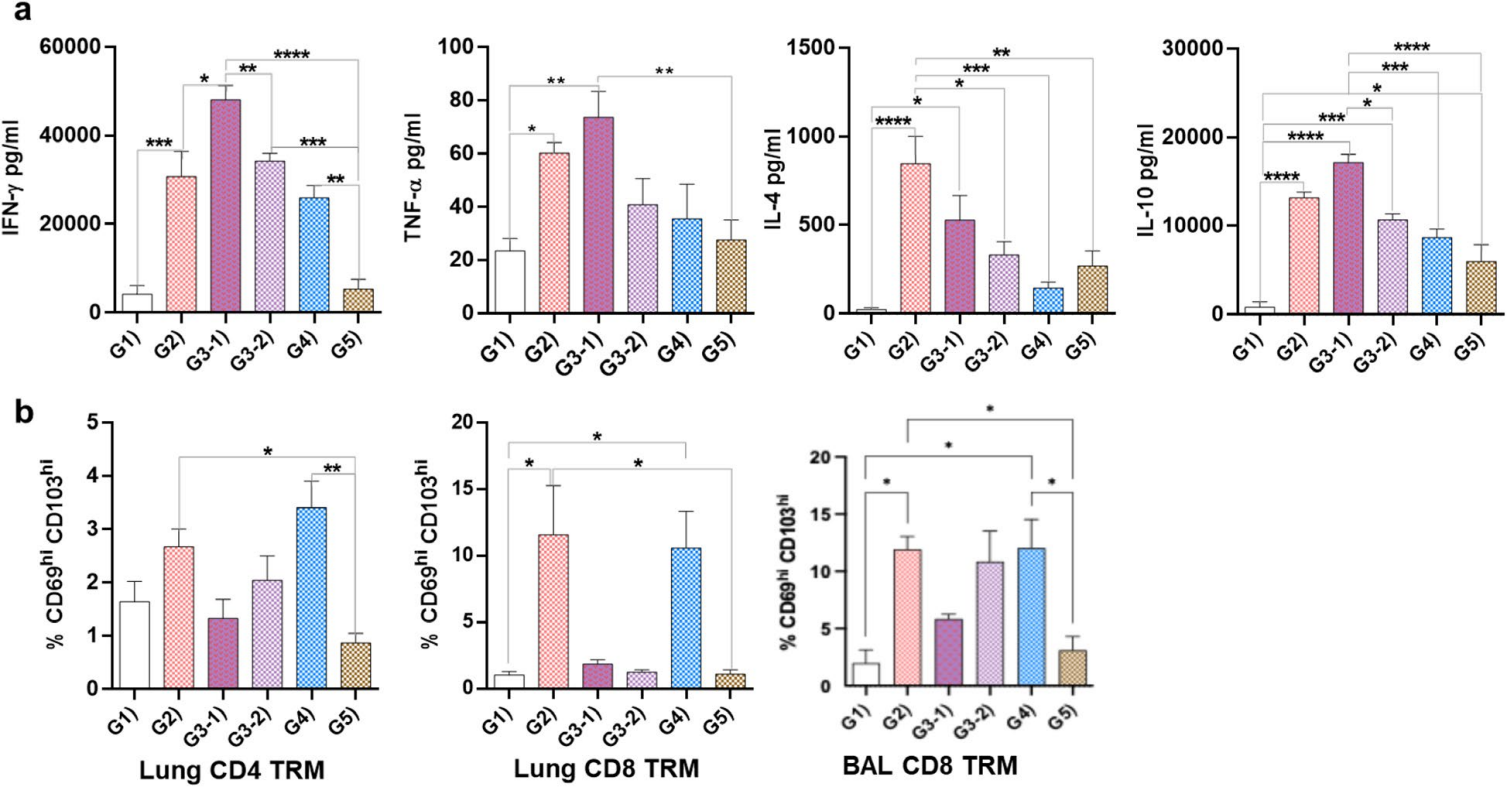
Cellular responses induced by SRBD‐PCF. (a) Secreted cytokines in splenocyte cultures after stimulation with mammalian‐derived RBD. (b) Detection of Lung CD4/CD8 TR M and BAL CD8 TRM after recall stimulation with mammalian‐derived RBD. The statistical significance was determined by one‐way ANOVA with Tukey's multiple comparisons test; all data are presented as means of triplicate technical repeats ±SE. *p ≤ 0.05, **p ≤ 0.01, ***p ≤ 0.001, ****p ≤ 0.0001.

On stimulation of splenocytes of immunised mice with RBD antigen, all groups responded with the production of IFN‐γ, apart from the PBS (G1) and RBD alone (G5) groups; however, the highest response was recorded for the group immunised twice systemically and once intranasally (G3‐1). A similar trend was also observed for TNF‐α, but with G2 and G3‐1 groups being the only ones statistically above the background levels (Figure 5a). As for the Th2 cytokines, all groups except G1 and G5 produced IL‐4, with G2 producing the highest levels, while G2 and G3‐1 produced the highest levels of IL‐10. Notably, the G2 group (SRBD‐mPCF systemic prime, mucosal boost) induced high levels of all four cytokines measured, thus indicating a mixed Th1/Th2 cellular response.

We also tested for the presence of antigen‐specific tissue resident memory (TRM) T cells in the lung tissue and BALF of immunised mice, using the gate strategy shown in Figure S4. We observed that CD4+ TRM T cell responses were generally muted, with only G2 and G4 showing a statistically significant increase compared to the RBD alone (G5) but not PBS (G1) group (Figure 5b). In contrast, we observed a high frequency of CD8+ TRM T cells in G2 and G4 groups, in both lung tissue and BALF (Figure 5b, middle and right panel), indicating a prominent CD8 T cell memory profile in the lungs following nasal boosting with SRBD‐mPCF.

### 3.7 Protection Against SARS‐CoV‐2 in hACE2 Transgenic Mice

**FIGURE 6:**
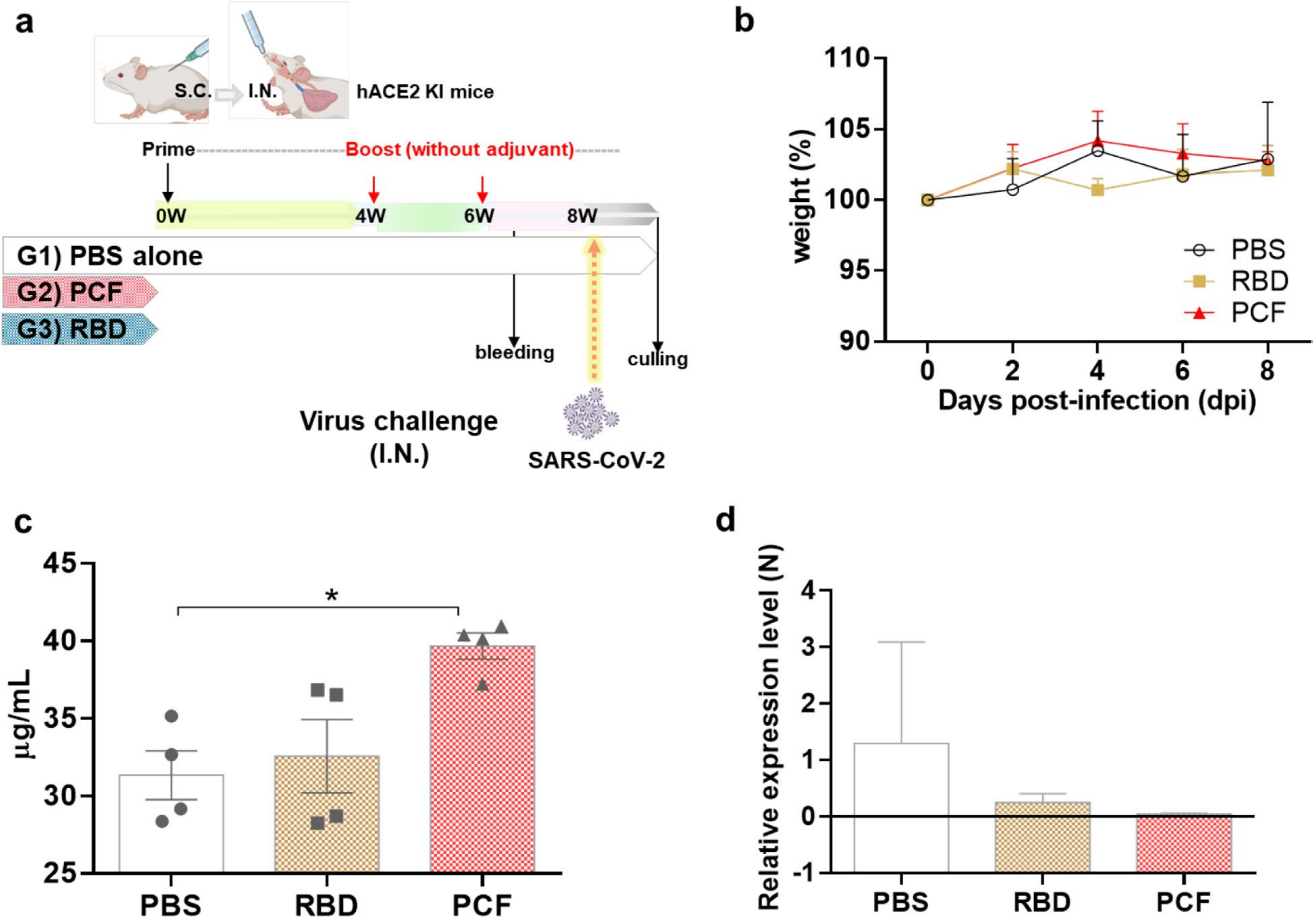
Immunisation and SARS‐CoV‐2 challenge in hACE2KI mice (a) Schedule for immunisation and viral challenge. Subcutaneous prime and nasal boost strategy used for vaccination before viral challenge with 2 × 105 PFUs SARS‐CoV‐2 (Wuhan strain): Priming with 50 μg of RBD formulated with 15 μg QuilA; boosting with 15 μg of SRBD‐PCF or 6 μg (molecular equivalent) of RBD without adjuvant for Groups 2 and 3, respectively. PBS only was used as a control group (b) the effects of viral challenge on body weight. The mice were weighed daily for 8 days after infection with SARS‐CoV‐2; no significant differences between groups were detected. (c) Quantification of RBD‐specific IgG antibody prior to viral challenge, using mouse IgG as a standard curve. (d) RNA expression for the N protein of SARS‐CoV‐2 in the lung detected using qRT‐PCR at 8 days post‐infection. Statistical analysis was performed using two‐way ANOVA followed by Tukey's correction test for multiple comparisons. The data are presented as mean ± SEM; *n* = 4. *p ≤ 0.05.

With the intention of modelling mucosal boosting of a preexisting but suboptimal systemic immunity to SARS‐CoV‐2, we performed a mouse pathogenic challenge study using transgenic mice expressing hACE2 receptor. Mice were systemically primed (subcutaneously) with RBD + Quil‐A, and subsequently twice nasally boosted with either PBS, SRBD‐mPCF, or RBD alone, before intranasal challenge with SARS‐CoV‐2 (Figure 6a). Mice weight was recorded over an 8‐week post‐infection period and no significant differences were observed among any of the three groups (Figure 6b). Upon cull, antigen‐specific IgG titres were measured in sera, showing that the SRBD‐mPCF group had the highest concentration though this was statistically significant only when compared with the PBS group (Figure 6c). Viral RNA was also assessed in the lung tissue as a proxy for viral load, revealing that only the PBS group had detectable RNA, whereas the SRBD‐mPCF and RBD groups had none (Figure 6d). Due to small sample size and large intragroup variability, it was unfortunately not possible to measure whether the observed difference in the viral load was statistically significant.

### 3.8 Feasibility of SRBD‐PCF as an Inhalable Vaccine

**FIGURE 7:**
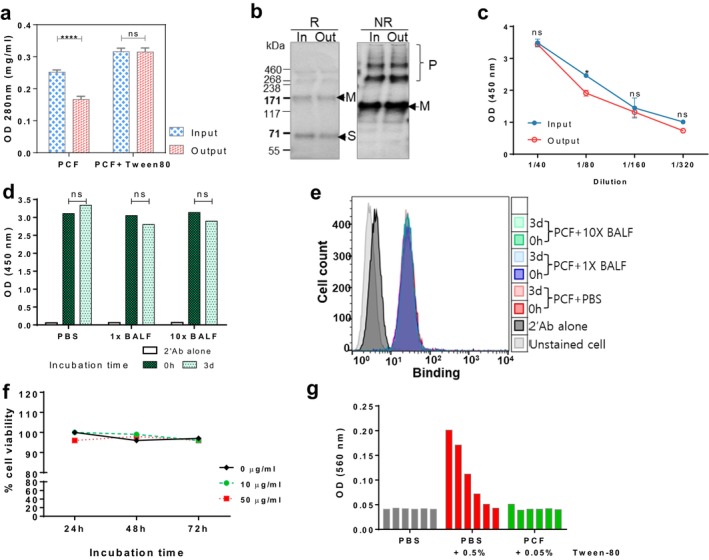
Feasibility of aerosolisation and safety profile of SRBD‐hPCF. (a–c) Aerosolisation of SRBD‐hPCF protein and its bio‐functionality after nebulising in Omron device. (a) Protein content recovery from condensate with/without 0.05% polysorbitol‐80. (b) SDS‐PAGE and immuno‐detection of SRBD‐hPCF before and after aerosolisation. NR, non‐reducing; R, reducing, ‘In’, input into nebuliser, ‘Out’, recovered from condensate. (c) GM1 binding activity. The statistically significant differences between input and output for all conditions were determined by two‐way ANOVA with Sidak's multiple comparisons test. *p* < 0.05 was considered significant. ‘ns’, non‐significant. (d–f) Test for stability of SRBD‐PCF (10 μg) after incubation with PBS, or 1× BALF or 10× BALF from NHP. (d) GM1 binding activity before and after 3‐day incubation with 1× or 10× NHP BALF. (e) APC binding activity before and after aerosolisation. (f) 72‐h U937 cells viability on incubation with SRBD‐PCF formulated in PBS containing 0.05% Tween‐80. (g) Measurement of hemolytic activity of SRBD‐hPCF with human red blood cells (RBC). 10 μg of PCF vaccine containing 0.05% Tween‐80 as compared to 0.5% Tween‐80 alone in a total 100 μL volume of RBC. The bars represent 1 in 2 serial dilutions.

To test the feasibility of human application by respiratory route, the stability of SRBD‐hPCF was analysed after aerosolisation and recovery from the Omron MicroAir nebuliser (Figure 7a). Aerosolisation of the protein alone resulted in a partial loss of protein, presumably due to denaturation and precipitation, but this was prevented by the addition of Tween‐80 as an excipient. SDS‐PAGE analysis and Coomassie staining of the recovered protein indicated no change in distribution between different structural forms, with monomers and polymers present in a similar ratio, before and after aerosolisation (Figure 7b). Likewise, GM1 binding was not affected by aerosolization (Figure 7c). To test the stability of SRBD‐hPCF in mucosal fluids, the protein was incubated with neat or 10× concentrated BALF from nonhuman primates from a different study (White et al. 2023), and subsequently its capacity to bind GM1 by ELISA (Figure 7d) and APC by flow cytometry (Figure 7e) tested. There was no detrimental effect of mucosal fluid treatment for 3 days on either activity. Finally, we tested if SRBD‐hPCF exhibited any potential cytotoxicity or haemolytic activity against human cells. In a cytotoxicity assay, increasing concentrations of SRBD‐hPCF were added to U937 monocytes and their viability was monitored over 72 h, with no detectable loss observed (Figure 7f). In a haemolytic assay with human red blood cells, SRBD‐hPCF formulated with 0.05% Tween‐80 did not cause cell lysis, which only partially occurred with a 10‐fold increase in detergent concentration (Figure 7g). Thus, in summary, we demonstrated that the vaccine can be aerosolised without loss of material or activity, is stable in a mucosal environment, and does not cause any cytotoxicity or haemolytic activity to human cells.

## 4 Discussion

In this study, we tested our newly developed self‐adjuvanting vaccine Platform CTB‐Fc (PCF) in the context of SARS‐CoV‐2 infection, with a view to mucosally boost pre‐existing but insufficient systemic immunity due to prior exposure or vaccination. Our findings indicate that these protein‐only polymeric complexes can be produced in a stable, functionally active form, which is retained after aerosolisation, are nontoxic to human cells and, importantly, that they are immunogenic in mice after nasal delivery and recognised by human immune cells and antibodies. While this vaccine construct can be expressed in mammalian cells, we opted for a plant expression system as the production platform of choice for these complex macro‐molecules.

### 4.1 Vaccine Strategies to Increase the Efficiency Against SARS‐CoV‐2

We have observed a decrease in COVID‐19 vaccine efficacy due to several variants including Alpha, Beta, Delta, and Omicron, which are primarily mutated in the S gene (Sachs et al. 2022). Variants such as the COVID‐19 strain B.1.617.2 (also called Delta) contain 18 novel mutations compared to the ancestral strain (Ren et al. 2022) increasing the transmission rate of the virus and its affinity to lung epithelial cells (Chavda and Apostolopoulos 2022). Similarly, even greater antigenic drift was seen with the Omicron variant, which has more than 50 known mutations (Chatterjee et al. 2023). This strongly suggests that the variants are evolving to evade the immunity provided by the vaccines themselves (Chen et al. 2022; Erkihun et al. 2024), and therefore, different vaccine approaches including not only revised antigenic targets but also different vaccine platforms and routes of delivery should be evaluated for better prevention of infection. The virus first enters the nose or mouth and replicates within epithelial cells of the nasopharynx, causing an upper respiratory tract infection (Li et al. 2023). It is crucial to target respiratory pathogens at the first line of defence to prevent infections and the spread of the virus from asymptomatic shedding (Russell et al. 2020). To generate immunity in the respiratory tract, vaccines should be administered by the respiratory route to induce resident memory T and B cells in the lungs and rapidly generate neutralising antibodies locally (Allie et al. 2019; Park et al. 2024). However, there has been only a limited success so far in developing such vaccines, and this may, we argue, be in part due to suboptimal vaccine uptake and immune cell engagement in the mucosae.

Our vaccine approach that directly targets FcγRs on mucosal APC aims to circumvent some of these issues to better engage the mucosal immune system. IgG‐Fc fusion proteins have emerged as an attractive vaccine platform (Kim, Mason, et al. 2024) and there have been several studies utilising different iterations of these for COVID‐19 vaccines (Dashti et al. 2024; Ehteshaminia et al. 2023; Lee et al. 2024; Liang et al. 2024; Liu et al. 2020; Sun et al. 2024), including those expressed in plants (Siriwattananon et al. 2021). For instance, Akston Biosciences conducted a study on a subunit vaccine called AKS‐452, which contains an RBD‐Fc fusion and was tested with or without adjuvant (ISA 720) in preclinical and phase II clinical studies, respectively (Alleva et al. 2021). Li and colleagues applied the IgG‐Fc fusion with the S protein of SARS‐CoV‐2 intranasally to target the neonatal Fc receptor (FcRn) (Li et al. 2023). The study found that FcRn effectively transported the S‐Fc antigen into the airway, offering protection against SARS‐CoV‐2 infection and transmission (Li et al. 2023). We recently reported on a novel PCF vaccine technology, demonstrating proof of concept against dengue (Kim, Vergara, et al. 2024), and here, we demonstrate its application in the context of a respiratory infection, specifically SARS‐CoV‐2. Our approach is designed to target FcγRs/FcRn on mucosal APC by size‐controllable macromolecular forms, rather than by a single chain or monomeric Fc fusion form commonly used in aforementioned studies. This, we postulated, would lead to more efficient vaccine uptake by APCs. Larger molecular assemblies enhance vaccine uptake by APCs due to several mechanisms (Bachmann et al. 1993; Kumar et al. 2015), including: (i) multimeric structures better mimic the size and geometry of pathogens than single proteins and are more readily internalised by phagocytic cells; (ii) increased molecular size improves the interaction with surface receptors such as FcγRs or complement receptors, thereby facilitating receptor‐mediated endocytosis; and (iii) larger assemblies provide a higher local density of antigenic epitopes, promoting more robust crosslinking of B cell receptors and enhancing immunogenicity.

### 4.2 PCF Vaccine Platform

PCF consists of three domains: two key molecules (CTB and Ig‐Fc) that create a multimeric scaffold and a target antigen, such as SRBD of SARS‐CoV‐2, in a single polypeptide chain, as depicted in Figure 1. The first step to macromolecule assembly is through the IgG‐Fc domain by S‐S bond in the hinge region, forming a homodimer, followed by the noncovalent assembly of the CTB pentamer, with the resulting complex displaying approximately 10 antigen molecules. The quaternary structure of IgG‐Fc is particularly important for its interactions with receptors. This is due to the unique organisation of the two constant domains (CH2 and CH3) of the two antibody heavy chains. Thus, to allow maximum structural flexibility, we extended Fc with the C‐terminal β‐strand of CH1 and the N‐terminus of the IgG1 heavy chain hinge region. This resulted in a predicted horseshoe‐like stable configuration of the fusion protein. The quaternary structure of IgG1 is further stabilised by N‐linked glycosylation, also increasing solubility (Tan et al. 2014). In a range of in vitro binding assays, we demonstrated that SRBD‐hPCF could bind to both human cell lines and primary tonsil cells.

The second key molecule in the PCF vaccine platform is the non‐toxic B subunit of CT, which serves both as a molecular adjuvant and an excellent delivery vehicle (Baldauf et al. 2015). CTB's capacity to polymerise provides the structural backbone to PCF macromolecules and our computational modelling of their structures predicted the correct configuration and receptor accessibility for each of the three vaccine components, Ig‐Fc, CTB, and SRBD. As the prediction of protein structure via AlphaFold modelling cannot always replace experimental structure determination (Terwilliger et al. 2024), we used several functional binding assays to demonstrate the correct structural configuration of PCF vaccine components. Thus, the Fc component's functionality was verified by APC and C1q binding assays, while CTB was tested in the GM1 ELISA assay. Since the only variable in the PCF construct is the antigen itself, it is important to also confirm antigen accessibility to immune cells, and particularly to B cells, which can only recognise unprocessed antigens. Furthermore, to induce relevant antibody responses, the antigen should be in the correct conformation. We therefore tested for this using several different assays, including binding to the ACE2 receptor on monocytes, and recognition and IC formation by immune sera from COVID‐19 patients and vaccines, with each assay confirming that SRBD is correctly folded and accessible within the PCF complex. SRBD‐PCF could also induce proliferation of CD4 T cells from pre‐exposed or vaccinated human subjects.

For intended aerosolised vaccine delivery, it was also important to demonstrate that the SRBD‐hPCF construct could be recovered after nebulisation and that it is not cytotoxic or hemolytic to human cells. This we showed by measuring protein yield after nebulisation and assessing various functional properties, with no significant differences observed between pre‐ and post‐nebulised fraction. Furthermore, the vaccine formulation was shown to be noncytotoxic and nonhemolytic towards human cells, paving the way for potential human application.

### 4.3 Immunogenicity and Protective Potential of SRBD‐PCF in Mice

We then performed a series of experiments to test the immunogenicity of SRBD‐mPCF in mice, using the mouse version of the construct (based on mouse IgG2a). In the initial experiments, we immunised wild type BALB/c mice systemically, then boosted nasally, with or without adjuvant. Our rationale for this vaccine regimen was to see if nasal boosting could induce both local immunity and enhance existing but insufficient systemic immunity, thus mimicking human hosts whose immunity after infection or immunisation has waned over time. Our findings suggest that indeed, SRBD‐mPCF could induce antigen‐specific IgA and predominantly CD8+ TRM T cells in the lungs, as well as boost systemic antibody and cellular immune responses. This was true whether SRBD‐mPCF was used on its own or when combined with Quil‐A adjuvant, though responses were generally somewhat higher in the presence of the adjuvant. We noted that SRBD‐mPCF induced a mixed Th1/Th2 T cell profile systemically, with significant levels of IFN‐γ, TNF‐α and (Th1), as well as IL‐4 and IL‐10 (Th2) all present after restimulation of splenocytes with antigen. A balanced cytokine profile is generally preferable over a strongly biased one because it allows the immune system to more rapidly adapt to varying conditions and fine‐tune its responses and mechanisms. The induced systemic antibodies could neutralise the SARS‐CoV‐2 pseudovirus in vitro in a dose‐dependent manner, while BALF from immunised mice also showed higher neutralising activity compared to PBS immunised mice, though no differences could be detected between different vaccination regimens due to small sample size.

We then tested a similar vaccine strategy in hACE2KI mice bearing human ACE2 receptor and thus susceptible to SARS‐CoV‐2 infection. Similarly to WT mice, these mice also showed antigen‐specific antibody titres in their sera. After intranasal challenge with wild type SARS‐CoV‐2 (Wuhan), mice were monitored over an 8‐day period, during which there were no observable clinical symptoms of the infection. Upon cull, viral RNA could be found in only 2 out of 4 animals in the control group but none in the immunised animals. Thus, despite the observed trend towards protection, we could not draw any definitive conclusion due to the small sample size and an apparent low infectivity of SARS‐CoV‐2 in these mice, as also observed in another study (Cho et al. 2024). Though we did not have access to those, the K18‐hACE2 mice are generally considered a better model than hACE2 KI mice because the hACE2 is overexpressed on epithelial cells throughout the body of these mice, unlike in hACE2 KI mice. This is due to the regulation of the epithelial cell‐specific human keratin 18 (K18) promoter, resulting in a more severe SARS‐CoV‐2 infection compared to hACE2 knock‐in mice (Dong et al. 2022). Further assessment of the protective potential of SRBD‐mPCF in better, more advanced animal models of infection will comprise the next stage of development and testing of this mucosal vaccine candidate for COVID‐19.

### 4.4 Plants as an Expression System for Macro‐Proteins

Complex molecules, ranging from self‐assembling structures of very large size to well‐defined pentameric or hexameric forms—such as recombinant IC and multimeric antibody‐based proteins—have been extensively evaluated in mammalian cells (Mekhaiel et al. 2011) but have also been successfully produced in plants, both in wild‐type and glyco‐engineered variants, as described in our recent review (Kim, Mason, et al. 2024). Notably, a previous iteration of recombinant IC in our dengue studies (D‐PIGS; expressed in glycoengineered ΔXF benthamiana plants) exhibited comparable functional properties in vitro and in vivo to their mammalian‐derived counterparts expressed in CHO cells, with yields of purified protein being 17 mg/kg fresh weight plants and 2.5 mg/L CHO culture supernatant, respectively (Kim et al. 2018). The current iteration, PCF, was recovered from plant extracts at a similar level for both dengue antigen (Kim, Vergara, et al. 2024) and SRBD‐PCF in the present study, at approximately 20 and 10 mg per kg of fresh weight leaf tissue, respectively. Like in a previous study by Arakawa et al. (1997) these outcomes were achieved using an ER retention strategy, which enhanced protein folding efficiency and minimised degradation by endogenous plant proteases. This approach enabled high‐level expression and proper assembly of the target proteins, while also improving glycan homogeneity (mannose 5–9) and effectively eliminating plant‐specific immunogenic glycans such as β(1,2)‐xylose and core α(1,3)‐fucose (Ko et al. 2003). Göritzer et al. (2025) demonstrated that ER engineering—via overexpression of CTP:phosphocholine cytidylyltransferase (CCT) to expand ER membranes in *N. benthamiana*—combined with chaperone co‐expression, significantly enhanced secretory IgA production, achieving yields of nearly 1 g/kg fresh leaf weight. Both plant and mammalian cell systems are therefore considered viable platforms to produce complex proteins, and with further optimisation, plant‐based expression systems may serve as a competitive alternative to mammalian cell platforms in terms of production cost, scalability, and biosafety (Schillberg et al. 2019).

In conclusion, to our knowledge, this is the first demonstration of a protein‐based vaccine candidate for COVID‐19 that does not require exogenous adjuvants to induce both local (lung) and systemic immune responses. The prospect of inhaled or nasal vaccination and cost‐effective production using plant platforms makes this approach amenable for further development and testing.

## Author Contributions

M.‐Y.K. conceived the concept, performed the cloning, expression, prediction of protein structure, analysis of antigen‐specific IgA and the majority of in vitro experimental analyses, and co‐wrote the manuscript; A.C.T. performed flow cytometry studies, ex vivo immune human serum studies, cytotoxicity in RBCs, and primary mouse immunisation studies; J.K. performed mouse immunisation for virus challenge and evaluation; H.S.A. and A.S. performed TMC studies by flow cytometry; L.B. performed ex vivo studies with immune human cells (T‐cell proliferation and DCs); E.J.V. performed confocal microscopy and assisted with primary mouse immunisation studies with A.C.T., M.B. and E.G. performed neutralisation assays with SARS‐CoV‐2 pseudovirus; K.G. provided mammalian‐derived RBD protein and assisted with SEC analysis; T.‐H.K. provided input on structural studies; J.K.C.M. enabled plant expression studies; Y.‐S.J. conceived the project with M.‐Y.K. and R.R. and contributed to analyses and writing of the manuscript; R.R. oversaw the project and co‐wrote the manuscript with M.‐Y.K. and Y.‐S.J.

## Conflicts of Interest

T.‐H.K. is the CEO of GeneCell Biotech company in Jeonju, Korea; L.B. was an employee of the company ImmunXperts (Belgium) when this work was performed; all other authors declare no conflicts of interest.

## Supporting information


Appendix S1.

## Data Availability

The data that support the findings of this study are available on request from the corresponding author. The data are not publicly available due to privacy or ethical restrictions.
