# Supplementary material for: Structural Design and Immunogenicity of a Novel Self‐Adjuvanting Mucosal Vaccine Candidate for SARS‐CoV‐2 Expressed in Plants
Source: Plant Biotechnol J. 2025 Jul 21;24(1):284–99. doi: 10.1111/pbi.70278 (PMC12854891; doi:10.1111/pbi.70278)
Supplement: Supplementary file 1 — Appendix S1. [file PBI-24-284-s001.docx]

**Structural design and immunogenicity of a novel self-adjuvanting mucosal vaccine candidate for SARS-CoV-2 expressed in plants**

Mi-Young Kim^1,2^, Andy Cano Tran^2^, Ju Kim^1^, Humblenoble Stembridge Ayuk^4^, Adam Sparrow^2^, Lorenzo Bossi^5^, Megan Brown^2^, Emil Joseph Vergara^2^, Kathrin Göritzer^2^, Elisabetta Groppelli^2^, Tae-Ho Kwon^3^, Julian K.C. Ma^2^, Yong-Suk Jang^1^* and Rajko Reljic^2^*

Address:

^1^ Department of Molecular Biology and the Institute for Molecular Biology and Genetics, Jeonbuk National University, Jeonju 54896, Republic of Korea

^2^ Institute for Infection and Immunity, City St George’s University of London

^3^ Gencellbiotech Inc., Wanjusandan 5-ro, Wanju, Republic of Korea

^4^ Master de Sciences, Technologies, Santé, Mention Biologie Moléculaire et Cellulaire, Université Claude Bernard Lyon 1, 69100, Villeurbanne, France

^5^ ImmunXperts SA, a Q² Solutions Company, Gosselies, Belgium

*Corresponding author: Rajko Reljic, rreljic@sgul.ac.uk

Institute for Infection and Immunity, St George’s University of London, UK

London SW17 0RE, UK

Tel: +208-725 0554

Fax: +0208-725 3487

*Corresponding author:

Yong-Suk Jang, [yongsuk@jbnu.ac.kr](mailto:yongsuk@jbnu.ac.kr)

Department of Molecular Biology, Jeonbuk National University

Jeonju 54896, Republic of Korea

Tel: +82-63-270-3338

Fax: +82-63-270-3345

**Key words**: cholera toxin B subunit (CT-B), immunoglobulin Fc, macro molecular adjuvant, SARS-CoV-2, mucosal vaccine platform, APCs targeting

**Supporting experimental procedures**

***Method S1: Programs used***

NCBI (https://www.ncbi.nlm.nih.gov/) and UniProt (https://www.uniprot.org/) for bioinformatics; ProtParam tool in Expasy (https://web.expasy.org/protparam/) for computation of various physical and chemical parameters; AlphaFold 2 via UCSF ChimeraX version 1.8.dev202403271703 (https://www.rbvi.ucsf.edu/chimerax/download.html) for prediction of protein and match making; Protein Data Bank in Europe (https://www.ebi.ac.uk/pdbe/) for reference protein structure; FlowJo Software v10.7.2 (https://www.flowjo.com/solutions/flowjo/downloads/previous-versions) for analysis of flow cytometry; Malvern Zetasizer Software v7.12 (https://www.malvernpanalytical.com/en/support/software-download) for protein size measurement; GraphPad Prism Software version 9.0.0. (https://www.graphpad.com/) for statistical analysis; NIS-Elements Viewer 4.2 (https://nis-elements-viewer.software.informer.com/4.2/#google_vignette) for image analysis; BioRender (https://app.biorender.com/biorender-templates) for schematic illustration of mouse immunisation schedule.

***Method S2: Coomassie staining and Western blotting***

Gel electrophoresis and immunoblotting were performed with the purified proteins under reducing (R) or nonreducing (NR) conditions using 4%–12% or 3-8% precast gels (Life Technologies). Following electrophoresis, the gels were stained by Coomassie Brilliant Blue G250 (Sigma-Aldrich) or subjected to Western blot analysis. The protein-transferred nitrocellulose membranes were probed with specific antibodies: for Fc using peroxidase-conjugated anti-mouse or human IgG antiserum (1:2500 dilution; The Binding Site) and for SRBD using anti-SARS-CoV-2 Spike antibody (1:2500 dilution; R&D system) followed by anti-mouse IgG (light chain specific) peroxidase-conjugated antiserum (1:2500, Jackson ImmunoResearch), respectively. CTB was detected with anti-CT polyclonal antibody (1:5000 dilution; Sigma-Aldrich) followed by anti-rabbit IgG peroxidase-conjugated antiserum (1:2500, Sigma-Aldrich). The washed blots were developed using the ECL Plus Western blotting detection system (GE Healthcare).

***Method S3: Molecular size measurement of SRBD-PCF scaffold by DLS and SEC analyses***

The particle size of SRBD-PCF was compared with commercial human serum IgG and colostrum IgA (both from Sigma-Aldrich) and IgM from human serum (Invitrogen), by measuring their dynamic light scattering (DLS) properties. For the measurement, various concentrations (1 ~ 4 mg/mL) of proteins including unfractionated or fractionated SRBD-PCF were prepared in 70μL of PBS and applied into the disposable UV micro cuvette (Brand UV cuvette micro) for Zetasizer Nano-ZS instrument (Malvern) measurements. SRBD-PCF protein was also subjected to size-exclusion chromatography (SEC) on a HiLoad 16/600 Superdex 200 column (GE Healthcare, USA) equilibrated with PBS pH 7.4 and connected to an ÄKTA pure (GE Healthcare, USA) FPLC system. The approximate size of SRBD-PCF fractionated by SEC was calculated by running protein standards (Sigma-Aldrich, gel filtration markers kit for protein molecular weights 29,000-700,000 Da).

***Method S4: Assays for complement C1q, GM_1_ ganglioside and ACE2 binding of SRBD-PCF***

To confirm binding capacity of SRBD-PCF for GM_1_ (monosialoganglioside GM_1_, Sigma-Aldrich) or complement human C1q (Calbiochem), or recombinant human ACE2 (HEK, Miltenyi Biotec), ELISAs were performed as described previously [1]. Briefly, the plates were coated with 5 μg/mL of C1q or GM_1_, or 1μg/ml of ACE2 in PBS buffer (pH 7.4), then incubated with 1 μg/ml of SRBD-PCF in 2-fold serial dilutions thereof. Blocking and detection of protein were same as described for Western blot analysis. To develop the peroxidase reaction, 50 μg/mL of TMB substrate solution (Bethyl Laboratories, Inc) was added and reaction stopped by the addition of 25 μl/well of 2 M H_2_SO_4_. Absorbance was measured at 450 nm using a Sunrise plate reader (Tecan, UK).

***Method S5: Assays for binding, uptake and internalisation of SRBD-PCF by APC***

To confirm the binding capacity of SRBD‐hPCF to Fcγ‐receptor bearing cells and visualize the antigen uptake and internalisation, human THP‐1 or U937 monocyte/macrophage cell lines (ATCC) were used. APC binding and internalisation assays were performed as described previously [1]. Briefly, 1~2 × 10^5^ cells/well were incubated with 10 or 50 μg/ml of SRBD-PCF or IgG (50 μg/ml) alone. Confocal images were generated using a Nikon A1R confocal microscope (Nikon).

***Method S6: Human tonsillar mononuclear cells (TMC) SRB-PCF binding assays***

For tonsillar mononuclear cell (TMC) culture, human tonsils were collected from eligible patients who underwent elective tonsillectomy, between 2017-2019 (before onset of COVID-19 pandemic) and subsequently TMC were isolated by Ficol gradient protocol, frozen and stored in liquid nitrogen. To culture TMC, RPMI 1640 medium supplemented with 10% FBS and penicillin/streptomycin and glutamine (all Sigma-Aldrich) was used. For cell staining, 2x10^6^ TMC in U-bottom plates were incubated with the fluorescently labelled cell surface-specific antibody cocktail including against CD3 (50 μg/mL, Biolegend), CD4 (13 μg/mL, Biolegend), CD14 (400 μg/mL, Biolegend) and CD19 (200 μg/mL, Biolegend) in PBS for 1 hour. Staining for expression of ACE2 and Fcγ receptors (CD64 and CD32) was then performed on gated TMC populations, with primary ACE2 mouse monoclonal antibody (1:200-1:800, Proteintech) or anti-human CD64 (0.5 μg/ml, Biolegend) or anti-human CD32 (0.5 μg/ml, Biolegend). After washing with PBS, the cells were stained with anti-mouse IgG FITC secondary antibodies and incubated for 30 min at room temperature. For SRBD-PCF binding, cells were incubated with 5 μg/ml of the protein, washed and incubated with mouse anti-human IgG FITC. The cells were washed twice and then resuspended in 500 μL PBS and analised with Cytoflex S flow cytometer (Beckman Coulter), using Fluorescence minus one (FMO) control for gating. 10,000 cells were acquired and analysed using Flow Jo V10 Software and values reported as Mean Fluorescence Intensity (MFI).

***Method S7: Feasibility of SRBD-PCF aerosolisation and mucosal stability***

The feasibility of SRBD-PCF aerosolisation and protein recovery was as previously described [2] Briefly, SRBD-PCF formulated in PBS alone or with 0.05 % Tween-80 buffer in 2 ml was loaded into OMRON MicroAIR nebuliser (U22, Japan) and following 1 min aerosolisation, condensate collected in 5ml of self-standing screw cap tube (Merk) with parafilm-sealing and subsequently analysed for protein content, C1q, GM_1_ and APC binding, as described above. To test stability of the protein in a mucosal environment, 10 μg/mL SRBD-PCF was incubated with either PBS or nonhuman primate-derived 1X or 10X concentrated BALF [3] for 0’ to 3 days at 37 ℃ and analysed by Coomassie staining or GM_1_ or APC binding as described above.

***Method S8: Analyses for cell toxicity***

To ensure no cytotoxic effects on cells, cell viability of U937 after incubation of SRBD-PCF (0, 10, 50 μg/ml) for 1 day to 3 days was assessed using an automated counter BIO-RAD(R) after staining with Trypan Blue. The haemolytic assay was performed to determine the safety profile of purified protein samples. Whole EDTA-treated blood was washed four times with PBS to remove lysed cells and debris by centrifugation at 500 rcf, 4°C for 10 minutes. The supernatant was aspirated, and RBC pellet was resuspended in PBS to its original volume. Finally, the washed RBCs were diluted 1:50 in PBS. Protein serial dilutions or positive control (0.5% Tween-80) were prepared in PBS (20 μl final volume) and added to 180 μl of washed RBC in V-bottom plates and incubated for 45 min at 37°C. The plate was then centrifuged at 500 rcf for 5 min at 4°C to pellet the red blood cells followed by transfer of supernatant into another 96-well flat bottom plate. The haemolytic activity was determined by measuring the absorbance of the supernatant at 560 nm using a Tecan INFINITE pro 2000 spectrophotometer.

***Method S9: Generation of human dendritic cells (DC)***

Monocytes were isolated from PBMCs using a MACS magnetic separation column (CD14 MicroBeads, Miltenyi) and purity was evaluated by CD14 FITC Miltenyi FACS staining (BD Fortessa X-20). Cells were then resuspended at 10^6^ cells/ml and plated into a tissue culture treated 12-well microplate in CellGenix DC medium (CellGenix) supplemented with Gentamycin, IL-4 and GM-CSF for 5 days. At day 5, cells were stained for FACS analysis with several DC activation markers in order to assess their immature dendritic cell (iDC) state (CD14-FITC-Miltenyi, CD40-BV510-Biolegend, CD80-BV421-BD, CD83-PE-Vio 770-Miltenyi, CD86-APC-Miltenyi, CD209-PE-Miltenyi and HLA-DR BUV395-BD). On the same day, iDCs were harvested and seeded in the same culture medium in a 48-well plate with SRBD-PCF at two different concentrations (PCF1= 5 µg/ml and PCF2= 20 µg/ml). Keyhole limpet hemocyanin (KLH, 25 µg/ml) was used as positive control. Every condition was supplemented with TNFα and IL-1β to promote DC maturation and cells incubated 24 h.

***Method S10: Detection of antigen-specific antibodies***

To detect antigen-speciﬁc IgG and IgA, the immune sera and BALF collected as described above were probed in serial 2-fold dilutions. As a coating antigen, 10 μg/ml of CTB (Sigma) and SRBD-mPCF were used for initial antibody responses and mammalian-derived recombinant RBD (50 μg/mL) was used for the final antibody responses. In some experiments, the actual concentrations of specific antibodies in sera were quantified from the ELISA results using standard curves, which were prepared by coating serial dilutions of normal mouse IgG

***Method S11: Analysis of splenocyte-produced cytokines by ELISA***

Cell supernatants from antigen recall assays were stored at −80°C prior to the quantification of IFN-ɣ, TNF-⍺, IL-4 and IL-10 using ELISA kits following the manufacturer’s instructions (ThermoFisher Scientific), with the sample dilution of 1:20 for IFN-ɣ and 1:5 for TNF-⍺, IL-4 and IL-10. The TMB substrate absorbance at 450 nm was acquired on a plate reader (Tecan) and cytokine concentrations in the supernatant were interpolated from a standard curve and multiplied by sample dilution factor.

***Method 12: qRT-PCR assay for viral quantification***

Lung specimens from hACE2 KI mice infected with SARS-CoV-2 were preserved at –80°C after being transferred to individual vials containing TRIzol reagent (Thermo Fisher Scientific). The lung tissues were ground and then processed via a total RNA prep kit (Biofact) to isolate the total RNA. The extracted RNA was converted to cDNA using a Reverse-Transcription Kit (Promega). Using the SsoAdvanced Universal SYBR Green Supermix (Bio-Rad) and a CFX Connect Real-Time System (Bio-Rad Laboratories), qRT-PCR for gene expression analysis was performed. Initial denaturation of the template was carried out at 95°C for 3 min, followed by 40 cycles of denaturation at 95°C for 15s, annealing at 52°C for 30s, and extension at 72°C for 30s. The primer sequences were as follows: N protein of SARS-CoV-2, forward 5′-ATG CTG CAA TCG TGC TAC AA-3′ and reverse 5′-GAC TGC CGC CTC TC-3′; mouse β-actin, forward 5′-CGT ACC ACA GGC ATT GTG A-3′ and reverse 5′-CTC GTT GCC AAT AGT GAT GA-3′. Using mouse β-actin as the endogenous control gene, the relative gene expression level was standardized. CFX Maestro software (Bio-Rad Laboratories) was used to perform fold-change calculations.

**References**

1. Kim, M.Y., et al., Marked enhancement of the immunogenicity of plant-expressed IgG-Fc fusion proteins by inclusion of cholera toxin non-toxic B subunit within the single polypeptide. Plant Biotechnol J, 2024. **22**(5): p. 1402-1416.

2. Tran, A.C., et al., Mucosal Therapy of Multi-Drug Resistant Tuberculosis With IgA and Interferon-gamma. Front Immunol, 2020. **11**: p. 582833.

3. White, A.D., et al., Spore-FP1 tuberculosis mucosal vaccine candidate is highly protective in guinea pigs but fails to improve on BCG-conferred protection in non-human primates. Front Immunol, 2023. **14**: p. 1246826.

**Supporting figures**


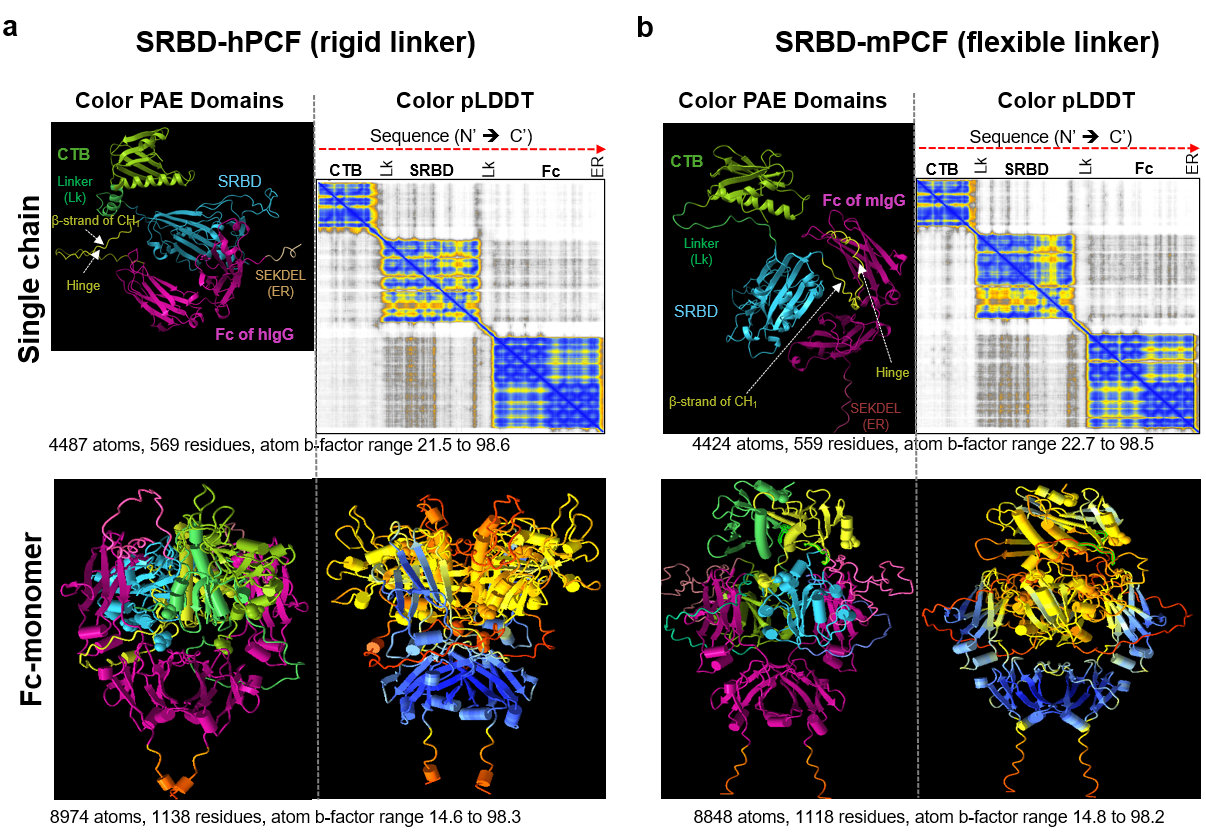


**Fig.S1 Accuracy of predicted protein structure. a)** Human PCF and **b)** mouse PCF. The accuracy is indicated with Color PAE (Predicted aligned errors) Domains showing in a different color of each domain, and Color pLDDT (predicted Local Distance Difference Test) corresponding to model confidence in each region. The pLDDT score ranges from 0 to 100: very high confidence in dark blue (pLDDT > 90); confident in light blue (90 > pLDDT > 70); low confidence in yellow (70 > pLDDT > 50); very low confidence in orange (pLDDT < 50). The b-factor is the atomic displacement or flexibility within a crystal structure of a protein, providing insight into the dynamic behaviour and stability of atoms within the protein. The lower end represents regions of the protein that are likely to be highly stable and less flexible and higher end represents regions that are somewhat flexible and possibly more involved in dynamic processes.


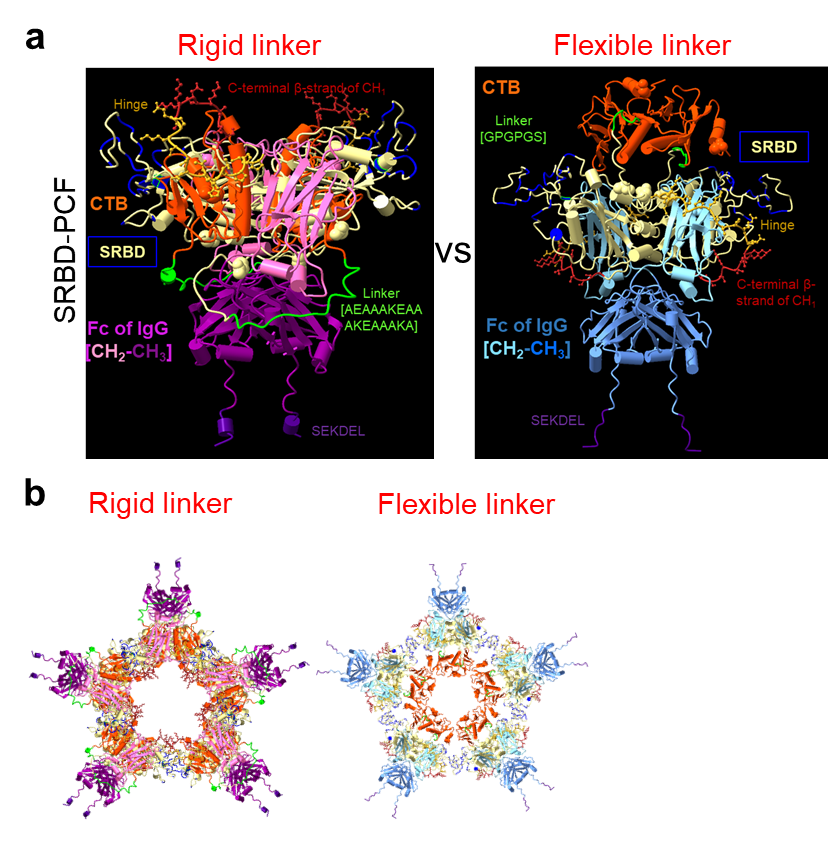


**Fig.S2 Prediction linker impact on structure. a)** Comparing linkers in human (rigid) and mouse (flexible) PCF. **b)** Diagram of putative CTB-pentamer generated with a predicted Fc-monomer**.**


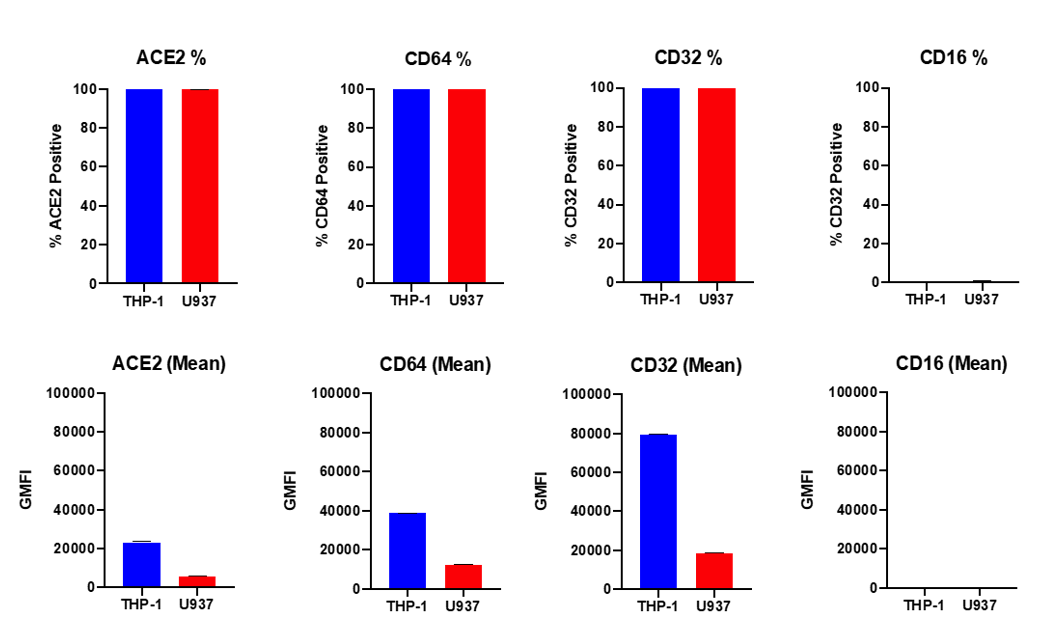


**Fig.S3 Expression of receptors on THP-1 and U937 cells.** Upper panels show percentages of cells expressing indicated receptor, whereas bottom panels show the relative levels of expression expressed as geometric mean fluorescence intensity (GMFI), as measured by flow cytometry.


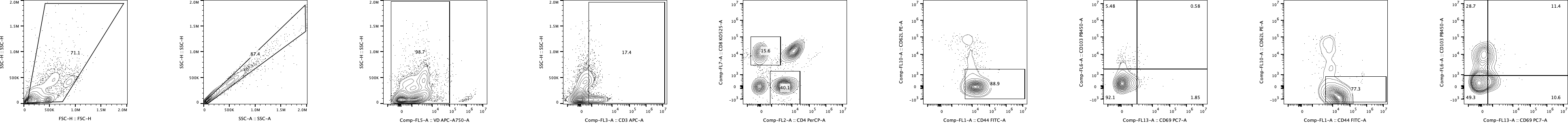


Cells

Single Cells

Viable

Cells

CD3

CD8

CD4

CD4 TEM

CD4 TRM

CD8 TEM

CD8 TRM


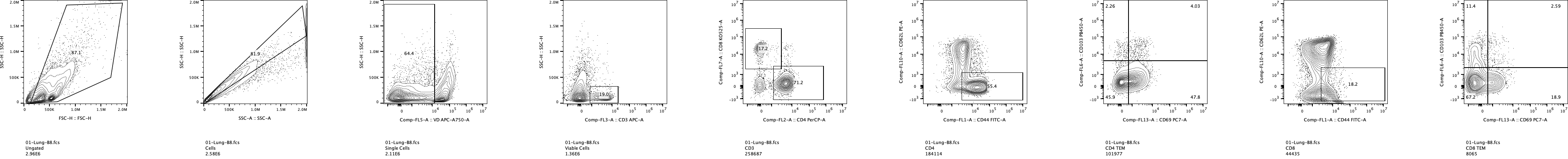


Cells

Single Cells

Viable

Cells

CD3

CD8

CD4 TEM

CD4 TRM

CD8 TEM

CD8 TRM

**Lung TRM**

**BAL TRM**

CD4

Gating strategy: Single Cells → Viable Cells → CD3 → CD4/CD8 → CD44^hi^ CD62L^lo^ → CD69^hi^ CD103^hi^

**Fig.S4** Gating strategy for lung CD4/CD8 TRM (upper panels) and BALF CD8 TRM (lower panels)
